# Identification of the *ALMT* gene family in the potato (*Solanum tuberosum* L.) and analysis of the function of *StALMT6*/*10* in response to aluminum toxicity

**DOI:** 10.3389/fpls.2023.1274260

**Published:** 2023-11-20

**Authors:** Feng Zhang, Sixia Jiang, Qiong Li, Zhiying Song, Ying Yang, Shirui Yu, Zongyue Nie, Moli Chu, Yanlin An

**Affiliations:** ^1^Department of Food Science and Engineering, Moutai Institute, Renhuai, Guizhou, China; ^2^Department of Brewing Engineering, Moutai Institute, Renhuai, Guizhou, China; ^3^Agriculture Science Institute of Bijie, Bijie, Guizhou, China; ^4^Anhui Provincial Key Laboratory of the Conservation and Exploitation of Biological Resources/College of Life Sciences, Anhui Normal University, Wuhu, Anhui, China

**Keywords:** StALMT family, potato, StALMT6, StALMT10, Al toxicity

## Abstract

**Introduction:**

Aluminum (Al)-activated malate transporters (ALMTs) play an important role in the response to Al toxicity, maintenance of ion homeostasis balance, mineral nutrient distribution, and fruit quality development in plants. However, the function of the *StALMT* gene family in potato remains unknown.

**Methods and results:**

In this study, 14 *StALMT* genes were identified from the potato genome, unevenly distributed on seven different chromosomes. Collinearity and synteny analyses of *ALMT* genes showed that potatoes had 6 and 22 orthologous gene pairs with Arabidopsis and tomatoes, respectively, and more than one syntenic gene pair was identified for some *StALMT* gene family members. Real-time quantitative polymerase chain reaction (qPCR) results showed differential expression levels of *StALMT* gene family members in different tissues of the potato. Interestingly, *StALMT1*, *StALMT6*, *StALMT8*, *StALMT10*, and *StALMT12* had higher expression in the root of the potato cultivar Qingshu No. 9. After being subjected to Al^3+^ stress for 24 h, the expression of *StALMT6* and *StALMT10* in roots was evidently increased, displaying their decisive role in Al^3+^ toxicity.

**Discussion:**

In addition, overexpression of *StALMT6* and *StALMT10* in Arabidopsis enhanced its tolerance to Al toxicity. These results indicate that *StALMT6* and *StALMT10* impart Al^3+^ resistance in the potato, establishing the foundation for further studies of the biological functions of these genes.

## 1 Introduction

Acidic soil accounts for 30%–40% of the world’s arable land, and this proportion is even higher in subtropical regions, reaching approximately 70% (Kochian et al., 2015). Previous studies have shown that aluminum (Al) toxicity in acidic soils is an important limiting factor affecting crop yield. It inhibits the growth of main root crops, promotes lateral root growth, hinders the absorption of water and other nutrients, reduces photosynthesis, and ultimately leads to crop yield reduction (Zheng, 2010; Magalhaes et al., 2018; Chen et al., 2022). The aluminum-activated malate transporter (ALMT) is a class of membrane proteins present in plants that plays a vital role in the plasma membrane transport of plant malic acid. The *ALMT* gene family has been identified and depicted in different plants and crops. For instance, 14 members of the *AtALMT* gene family have been identified in the Arabidopsis genome (Kovermann et al., 2007). In the soybean genome, 34 members of the *GmALMT* gene family have been identified, and studies have demonstrated that GmALMT5 is a plasma membrane protein that mediates malate efflux from the roots (Peng et al., 2018). In sugarcane, 11 members of the *ALMT* gene family were recognized, and six of these genes played a role in Al toxicity (Ribeiro et al., 2021). Additionally, the *ALMT* gene family in Chinese white pear and apple was identified, which plays a key role in various physiological functions such as malate accumulation and organic acid efflux (Lin et al., 2018; Ma et al., 2018). In hydrangea (*Hydrangea macrophylla* [Thunb.] Ser.), 11 *ALMT* gene family members were recognized based on transcriptome data. Furthermore, the expression of three genes (*HmALMT5*, *HmALMT9*, and *HmALMT11*) in yeast increased tolerance to Al toxicity (Qin Z. et al., 2022).

The main functions of the ALMT family include tolerance to Al toxicity, regulation of stomatal resistance, transportation of mineral nutrition, microbial interactions, and seed development (Sharma et al., 2016). *TaALMT* was the first Al-activated malate transporter gene discovered and identified in wheat (*Triticum aestivum* L.). The main function of *TaALMT1* is to enable the transport of malate to bind with Al ions outside the cell, preventing Al ions from entering the root-tip cells (Sasaki et al., 2004). Subsequently, *AtALMT1* (Hoekenga et al., 2006), *BnALMT1* and *BnALM2* (Ligaba et al., 2006), *ScALMT1* (Collins et al., 2008), *ZmALMT1* (Piñeros et al., 2008) and *ZmALMT2* (Ligaba et al., 2012), *GmALMT1* and *HlALMT1* (Chen et al., 2013), *MsALMT1* (Chen et al., 2013), *SlALMT9* (Ye et al., 2017), and *BoALMT1* (Zhang et al., 2018) were also cloned. In Zea mays, *ZmALMT1* plays a significant role in mineral nutrition and ion homeostasis modes, rather than controlling a distinctive Al-activated citrate excretion reaction in the root (Piñeros et al., 2008). *ZmALMT2* is not related to Al stress but plays a significant role in mineral nutrient absorption and allocation (Ligaba et al., 2012). Interestingly, *AtALMT1* is responsible for mediating Al-activated malate secretion and responding to multiple signals indicating indole-3-acetic acid (IAA), abscisic acid (ABA), low pH, and hydrogen peroxide (Kobayashi et al., 2013). Furthermore, *HvALMT1* has been shown to be unrelated to Al resistance in barley (*Hordeum vulgare*) and functions as an anion channel to promote organic anion transportation in stomatal function and expanding cells (Gruber et al., 2010). Other members of the *ALMT* gene families code for anion channels, but the effects of substrate specificity, plasma membrane presence, and the dynamic balance of different functions including malic acid, osmotic regulation, and function of guard cells have significant differences (Kovermann et al., 2007; Xu et al., 2015).

There are 16 *ALMT* gene family members identified and analyzed in the tomato plant, which belongs to the *Solanaceae* family along with the potato. *SlALMT4* and *SlALMT5* are localized in the vascular bundles, and these two proteins secrete malate efflux (Sasaki et al., 2016). *SlALMT9* (Solyc06g072910–Solyc06g072920) was included in a genome‐wide association study with 272 tomato accessions, demonstrating that tonoplast‐localized was responsible for deciding the malic acid content of the fruit (Ye et al., 2017). Furthermore, the tomato-response Al toxicity and jasmonic acid (JA)-regulated mechanism revealed that *SlALMT3* is responsible for the regulatory mechanism underlying the crosstalk between Al and JA, and six *SlWRKYs* may act as upstream regulators of *SlALMT3* in this crosstalk response (Wang et al., 2020). In addition, *ALMT* (Solyc01g096140.3) was reported to be negatively correlated with the sugar/organic acid ratio in the tomato fruit (Li et al., 2021). Next, *SlALMT15* was confirmed to be related to the stomatal density of leaves and further affects photosynthesis and drought tolerance in the tomato (Ye et al., 2021). Finally, *SlALMT11* expressed in tomato leaf guard cells was associated with external malate-induced stomatal closure, but not with abscisic acid (Sasaki et al., 2022).

The edible part of the potato is in direct contact with the soil, and it is also the first contact site for high Al toxicity. High Al toxicity on potato growth and yield refers to the negative impact of excessive Al content in the soil on the growth and yield of potato crops. Al ions in the soil can harm the root system and reduce the uptake of essential nutrients by the potato plants. This stress can lead to stunted growth, reduced tuber formation, and lower yields. The function of the *StALMT* gene family in the potato remains correspondingly unknown. In this study, we identified and analyzed members of the *StALMT* gene family from the potato genome. Our study focused on chromosome distribution, gene nomenclature, physicochemical properties, subcellular localization, evolutionary relationship, and analysis of cis-acting elements of the members of the *StALMT* gene family. Additionally, we researched the expression of the *StALMT* gene family, the response of the potato cultivar to Al3^+^ toxicity, the relationship between malate secretion in the potato and Al toxicity, and the role of *StALMT6* and *StALMT10* in response to Al toxicity.

## 2 Materials and methods

### 2.1 Plant materials and growth conditions

The potato cultivar ‘Qingshu No. 9’ was used to analyze Al toxicity. Qingshu No. 9 was obtained from the Institute of Vegetables and Flowers, Chinese Academy of Agricultural Sciences. After germinating at 22°C, pre-elite seeds were transferred to 1/4 potato Murashige and Skoog (MS) basal salts with vitamins (Coolaber) non-buffered solutions at an initial pH of 5.5. The potatoes were grown at 25°C in growth chambers with a 12-h light/12-h dark photoperiod.

*Arabidopsis thaliana* Columbia-0 (Col-0) was obtained from the School of Life Sciences, Nanjing University. For growth experiments, Col-0 seeds were sterilized with 10% sodium hypochlorite for 4 min, washed in double-distilled water four times, and sown on MS or 1/4 MS medium containing 1% sucrose (Aldrich-Sigma) and solidified with 1% agar (Aldrich-Sigma). Our previous study specified the experimental methods for Al-tolerant growth phenotypes in Arabidopsis [30]. The plates were incubated at 4°C in darkness for 2 days and then positioned vertically. On day 10 after germination, the seedlings were imaged to measure primary root lengths using ImageJ (http://rsb.info.nih.gov/ij/) or were collected to weigh the biomass.

### 2.2 Identification and analysis of the ALMT gene family

The protein sequence of the reference genome “PGSC DM v4.03” was obtained from the Spud DB database (http://spuddb.uga.edu/). Then, the members of the potato *ATML* gene family were identified by gene annotation and BLASTp homology alignment. The MW and pI values of the ALMT family proteins were predicted using the ExPASy website (https://www.expasy.org/). Furthermore, the cis-acting elements were predicted by PlantCARE online software (https://bioinformatics.psb.ugent.be/webtools/plantcare/html/). Subsequently, the structural features of all candidate *ALMT* genes, i.e., protein motifs and cis-acting elements in the promoter region, are displayed using TBtools. Subcellular localization prediction of the StALMT family proteins was performed using WoLF PSORT (https://wolfpsort.hgc.jp/) (Horton et al., 2007). Both SOPMA database (https://npsa-prabi.ibcp.fr/cgi-bin/npsa_automat.pl?page=/NPSA/npsa_server.html) and Phyre2 (http://www.sbg.bio.ic.ac.uk/phyre2/html/page.cgi?id=index) software were used to predict the secondary and tertiary structures of the proteins, respectively (Gasteiger et al., 2005; Kelley et al., 2015).

### 2.3 Phylogenetic analysis and motif identification

All protein sequences were aligned using ClustalW in MEGA 7, and then the neighbor-joining (NJ) phylogenetic tree was constructed based on Poisson model with a bootstrap value of 1,000. All protein sequence motifs were predicted using MEME (http://meme-suite.org/) (Lu et al., 2021).

### 2.4 Chromosomal distribution, gene duplication, and synteny analysis

The location information of all gene family members is obtained from the GFF file of reference genome v4.03, and then the distribution of these genes on chromosomes is drawn based on the online web service of MG2C (http://mg2c.iask.in/mg2c_v2.1/). Using the multicollinearity scanning function module built in the TBtools software, gene duplication and collinearity analysis were completed and visualized (An et al., 2022).

### 2.5 RT-PCR and qRT-PCR

Total RNA was extracted from potato and Arabidopsis roots using TRIzol reagent (Invitrogen), followed by synthesis of poly(dT) complementary DNA using M-MLV Reverse Transcriptase (Promega). Reverse transcription–quantitative PCR (RT-qPCR) was performed using the SYBR Green I Master kit (Roche Diagnostics) according to the manufacturer’s instructions on a CFX Connect Real-Time System (Bio-Rad). Primer Premier 5.0 was used to design RT-qPCR primers for 14 members of the StALMT gene family, and Actin (PGSC0003DMT400047481) was used as the internal reference gene in PCR analyses. All primers used are listed in **Supplementary Table S2**. Three biological replicates were performed for each sample, and differential expression was calculated using the 2 [-Delta Delta C(T)] method (Livak and Schmittgen, 2001).

### 2.6 Cloning of the full-length CDS sequence of the StALMT6 and StALMT10 genes

The Qingshu No. 9 root was selected for the extraction of RNA material, and total RNA was extracted using the extraction kit [Tiangen Biochemical Technology (Beijing) Co., Ltd.]. Subsequently, cDNA was synthesized using reverse transcription. PCR amplification was performed using the cDNA as a template. The primers used were St-ALMT6-F1, St-ALMT6-R1, St-ALMT10-F1, and S-tALMT10-R1 (**Supplementary Table S1**). Amplified products were separated by electrophoresis using 1% agarose gel. The *StALMT6* and *StALMT10* fragments were tested for concentration after recovery using DNA Gel Extraction Kit. The recovered products were tailed with polyA using Taq enzyme and then ligated to the pMD 19-T vector and transformed into Escherichia coli DH 5 α competent cells. Growth and culture were coated on screening medium containing antibiotics. Finally, monoclonal colonies were picked for PCR, corrected and verified by sequencing.

### 2.7 Overexpression vector construction and Arabidopsis transformation

To construct expression vectors for the *StALMT6* and *StALMT10* transgenic expression vector, *StALMT6* and *StALMT10* CDSs were amplified from cDNA of Bishu No. 4 roots. For the Arabidopsis transformation, *StALMT6* and *StALMT10* sequences were cloned into the NcoI/SpeI sites of the plant expression vector pCAMBIA1302, and the plant overexpression vectors for pCAMBIA1302-35S-StALMT6 and pCAMBIA1302-35S-StALMT10 were obtained. The above obtained carriers were transferred into *Agrobacterium tumefaciens* strain GV3101, and Arabidopsis Col-0 plants were transformed by the floral dip method (Clough and Bent, 1998). Seeds were sterilized and then sown on solid 1/2 MS medium containing 35 μg mL^–1^ kanamycin. After 4 days of cultivation at 4°C, the seeds were transferred to a growth chamber with a light cycle of 16 h of light and 8 h of darkness at 25°C for 8 days. Subsequently, the plants were transplanted into soil. Specific primers (**Supplementary Table S2**) were designed for PCR identification of transgenic Arabidopsis to screen for positive plants. Three overexpressing homozygous T3 transgenic lines (OE strains) were obtained for each gene, and the best expressing OE strain was selected for subsequent Al toxicity experiments.

### 2.8 Morin fluorescence staining and measurements of Al contents by ICP-MS

Morin is a naturally occurring flavonoid compound derived from plants. It has the remarkable ability to form a fluorescent chelator when combined with aluminum ions, making it an ideal tool for aluminum ion fluorescence staining imaging. The method for morin fluorescence assay was previously described in our research (Zhang et al., 2019). In short, 7-day-old seedlings were incubated in 1/2 MS basal salts with vitamin solutions at pH 4.5, supplemented with 0 or 300 µM Al_2_(SO_4_)_3_ for 6 h. They were then stained with 1/6 MS solutions, without Al^3+^ (pH 4.5), containing 100 µM morin (Aldrich-Sigma) for 30 min. After rinsing with water thrice, the root tips were observed under a microscope (BX53, Olympus).

Three-week-old Arabidopsis seedlings were transferred onto 1/6-strength MS solutions (pH 5.0) without or with 300 µM Al_2_(SO_4_)_3_. After a 72-h treatment, Col-0, OE-*StALMT6-#2*, and OE-*StALMT10-#3* seedlings were collected and pooled into roots and shoots. After washing with distilled water three times, the samples were dried for 48 h at 100°C, milled to fine powder, weighed, and digested with concentrated HNO_3_ (Aldrich-Sigma) in a digester (DigiBlock ED16, LabTech). Ion concentration was measured by inductively coupled plasma mass spectrometry (ICP-MS; NexION 300, Perkin-Elmer). Each sample was tested at least three times.

### 2.9 Measurements of malate content

Root malate exudation was detected as previously described (Ding et al., 2013). Briefly, 3-week-old Col-0, OE-*StALMT6-#2*, and OE-*StALMT10-#3* seedlings were transferred onto 400 mL 1/6 MS basal salts with vitamin solutions (pH 5.0) without or with 300 µM Al_2_(SO_4_)_3_. After 3 days of incubation, the solutions were collected and passed through cation and anion exchange columns filled with 8 g resin-001X and 5 g DOWEX 1 × 8 chloride form (100–200 mesh, Aldrich-Sigma). After eluting with 10 mL of 1 M hydrochloric acid, the elate was concentrated in a rotary evaporator at 40°C. The residue was redissolved in 1 mL deionized ultrapure water and was used to detect malate and citrate concentration by high-performance liquid chromatography (HPLC; Model 1200SL, Agilent Technologies).

### 2.10 Statistical analysis

For all experiments in this paper, we performed at least three independent replications. The data obtained were statistically analyzed using the Student’s t-test (p < 0.05).

## 3 Results

### 3.1 Analysis of the StALMT family

**Figure 1:**
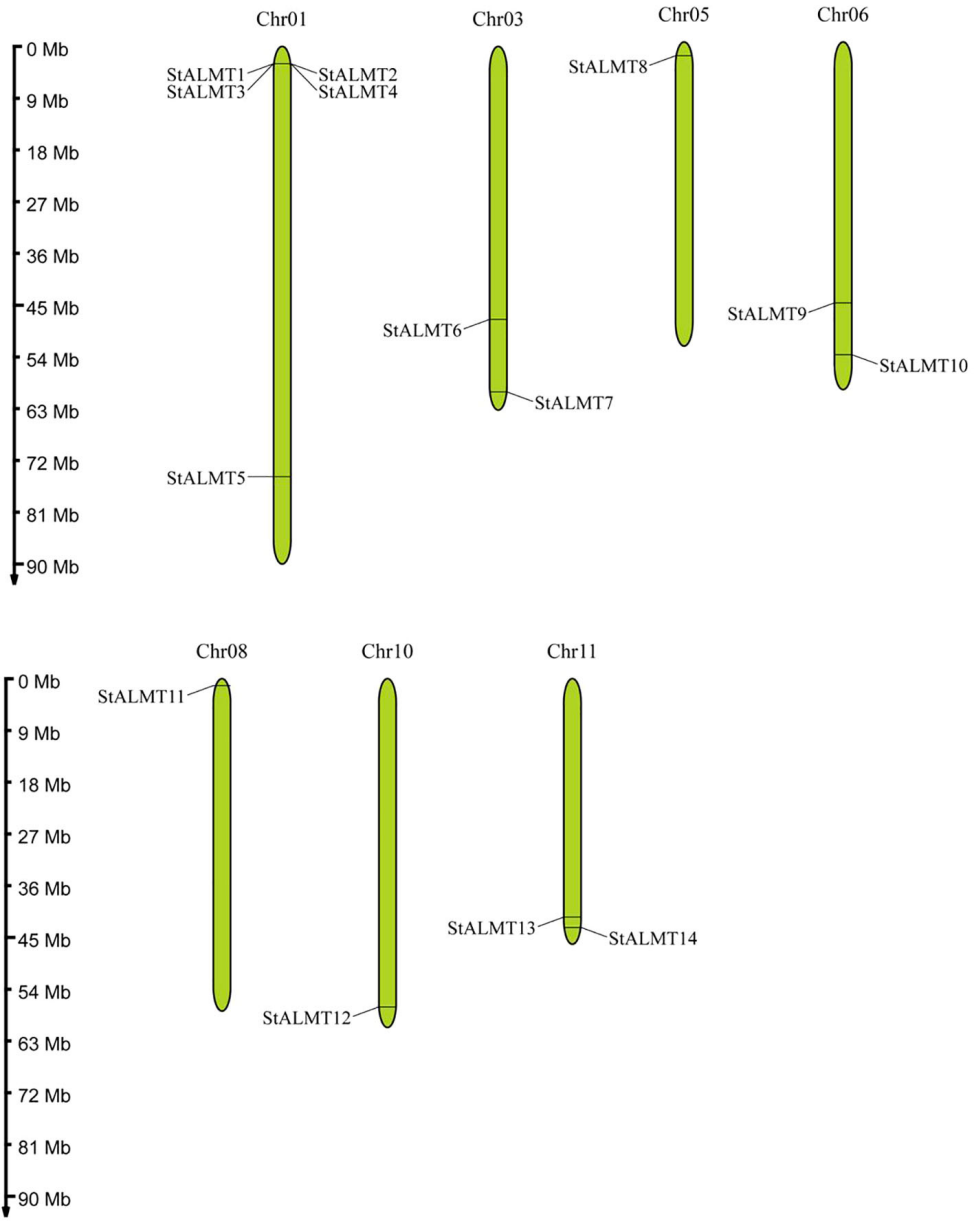
Chromosomal distribution of *StALMT*s. The scale bar on the left represents the length (Mb) of the potato chromosomes, and *StALMT*s are marked on both sides of the respective chromosome.

A study showed that Arabidopsis has 14 members of the ALMT family (Kovermann et al., 2007). The tomato and potato, which belong to the *Solanaceae* family, have 16 members of the ALMT protein family (Sasaki et al., 2016). In order to ascertain the number of ALMT protein family members in the potato, we used the information from the potato ALMT protein family in NCBI and Spud DB databases to obtain the sequence of StALMT proteins and related homologous proteins (Xu et al., 2011). A total of 14 ALMT family member proteins were identified in the potato, which were named StALMT1~14 based on their location on the chromosome (**Supplementary Table S1**). StALMT proteins were found to be 310~665 amino acids long and contain 3–7 transmembrane domains, and subcellular localization prediction showed that StALMT proteins are well distributed in the plasma membrane (Plas), endoplasmic reticulum (ER), cytolysosome (Cyto), chloroplast (Chlo), vacuole (Vacu), Golgi complex (Golg), and mitochondria (Mito). Interestingly, *StALMT1* is localized on the cytolysosome; StALMT2, StALMT4, StALMT6, and StALMT9 are localized on the plasma membrane; StALMT3 is localized in the vacuole; StALMT12 is localized in the chloroplast; StALMT7, StALMT8, and StALMT11 are distributed in both the plasma membrane and endoplasmic reticulum; StALMT10 and StALMT14 are localized on both the plasma membrane and vacuole. However, StALMT5 is distributed across the cytolysosome, endoplasmic reticulum, and mitochondria; StALMT13 is distributed across the plasma membrane, Golgi complex, and vacuole. Furthermore, as shown in **Figure 1**, these proteins are unevenly distributed on seven different chromosomes. Among them, chromosome 1 contains five StALMT members (StALMT 1~5), while chromosomes 3 (StALMT 6, 7), 6 (StALMT 9, 10), and 11 (StALMT 13, 14) all contain two StALMT members.

### 3.2 Prediction and analysis of the spatial structure of the StALMT protein family members

**Figure 2:**
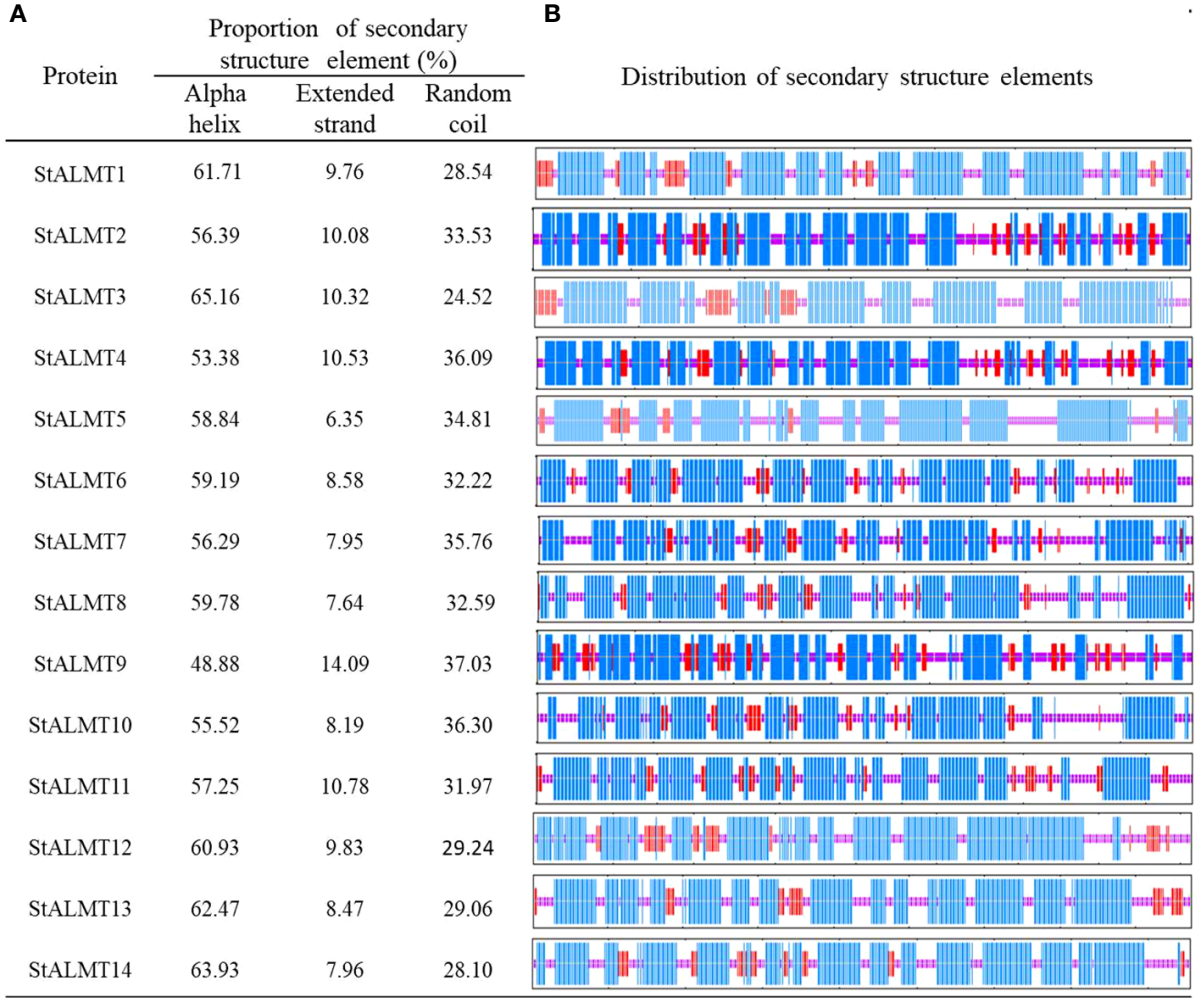
The secondary structure properties of the StALMT family members. **(A)** The proportion of the three protein secondary structure units in each StALMT family member. **(B)** The distribution of the three protein secondary structure units in each StALMT family member. The blue line in the figure indicates the alpha helix, the purple line indicates the random coil, and the red line indicates the extended chain.

**Figure 3:**
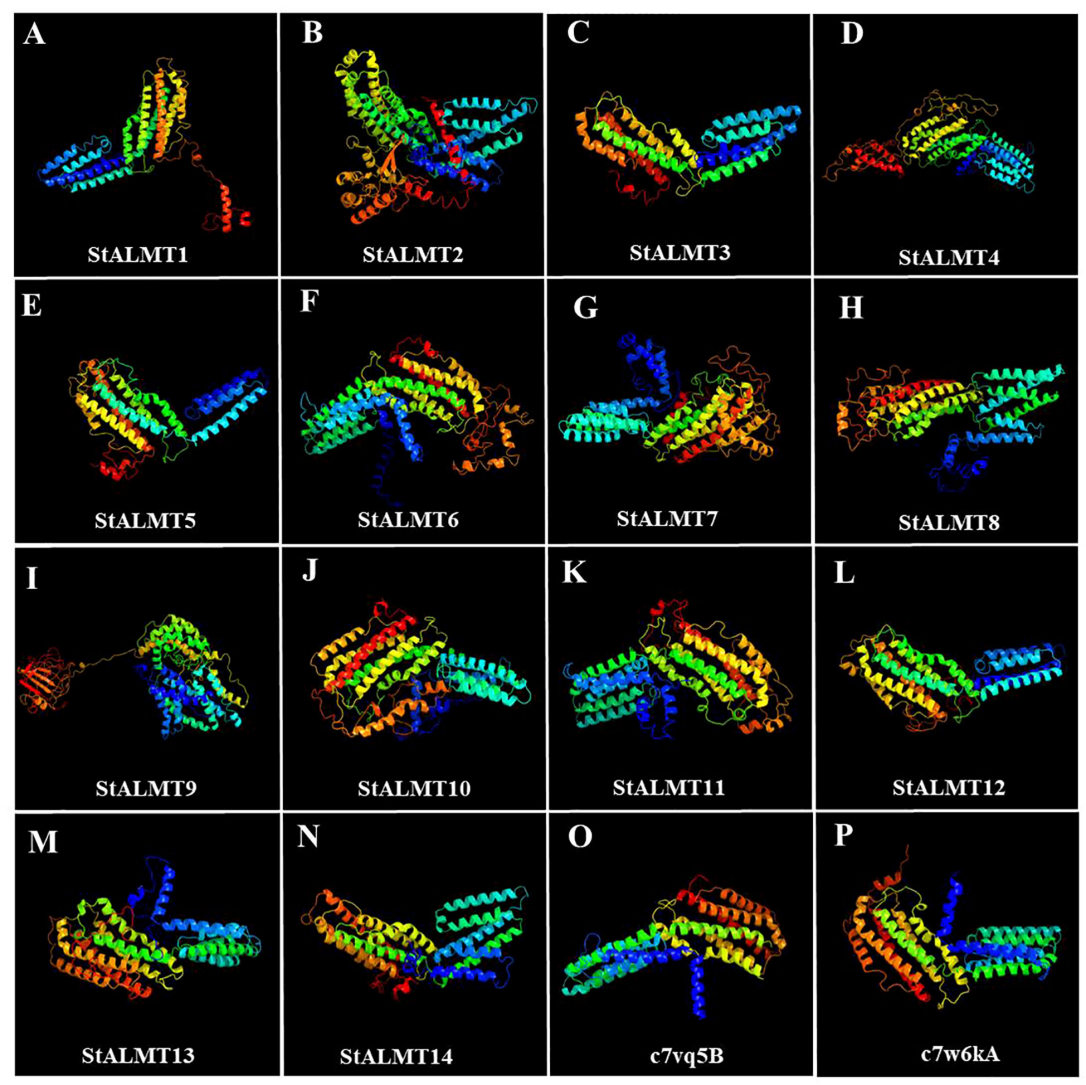
The tertiary structure properties of the StALMT family members. Tertiary structure of **(A)** StALMT1, **(B)** StALMT2, **(C)** StALMT3, **(D)** StALMT4, **(E)** StALMT5, **(F)** StALMT6, **(G)** StALMT7, **(H)** StALMT8, **(I)** StALMT9, **(J)** StALMT10, **(K)** StALMT11, **(L)** StALMT12, **(M)** StALMT13, **(N)** and StALMT14. **(O)** Cryo-EM structure of AtALMT1 with ID number c7vq5B derived from the fold library. **(P)** Cryo-EM structure of the GmALMT12/QUAC1 anion channel with ID number c7w6kA derived from the fold library.

To further investigate the StALMT protein family, we have predicted its secondary structures. Secondary structure analysis of StALMT proteins showed that all proteins comprised three structural units: α-helix, random coil, and extended strand, but the percentage and order distribution of these three structural units in these proteins were different (**Figure 2**). The highest percentage of α-helix was from 48.88% to 65.16%, followed by random coils from 24.52% to 37.03%. The percentage of the extended chains in each protein varied from 6.35% to 14.09%. Interestingly, the StALMT3 had the highest percentage of α-helices and the lowest percentage of random coils among all of the StALMT proteins. StALMT9 had exactly the opposite percentage of α-helices and random coils as StALMT3. The highest percentage of the extended strand was in StALMT9 and the lowest was in StALMT5 (**Figure 2A**). In addition, based on the publicly reported cryo-electron microscopy (cryo-EM) structure of AtALMT1 (c7vq5B)and GmALMT12 (c7w6Ka) (Qin L. et al., 2022; Wang et al., 2022), we predicted the tertiary structure of the StALMT protein family by using Phyre2 online software. The results showed that the tertiary structures of StALMT6, StALMT10, and StALMT11 were similar to that of Arabidopsis AtALMT1 (ID:c7vq5B) (**Figures 3F, J, O**). On the other hand, the tertiary structures of StALMT7 and StALMT13 were more similar to that of soybean GmALMT12 (c7w6kA) (**Figures 3G, M, P**). These predicted results provide a basis for further studying the functions and mechanisms of StALMT6 and StALMT10, and in the future, these predictions can be validated through experiments to further elucidate the relationship between their structure and function.

### 3.3 Phylogenetic and syntenic analysis of the potato StALMT proteins

**Figure 4:**
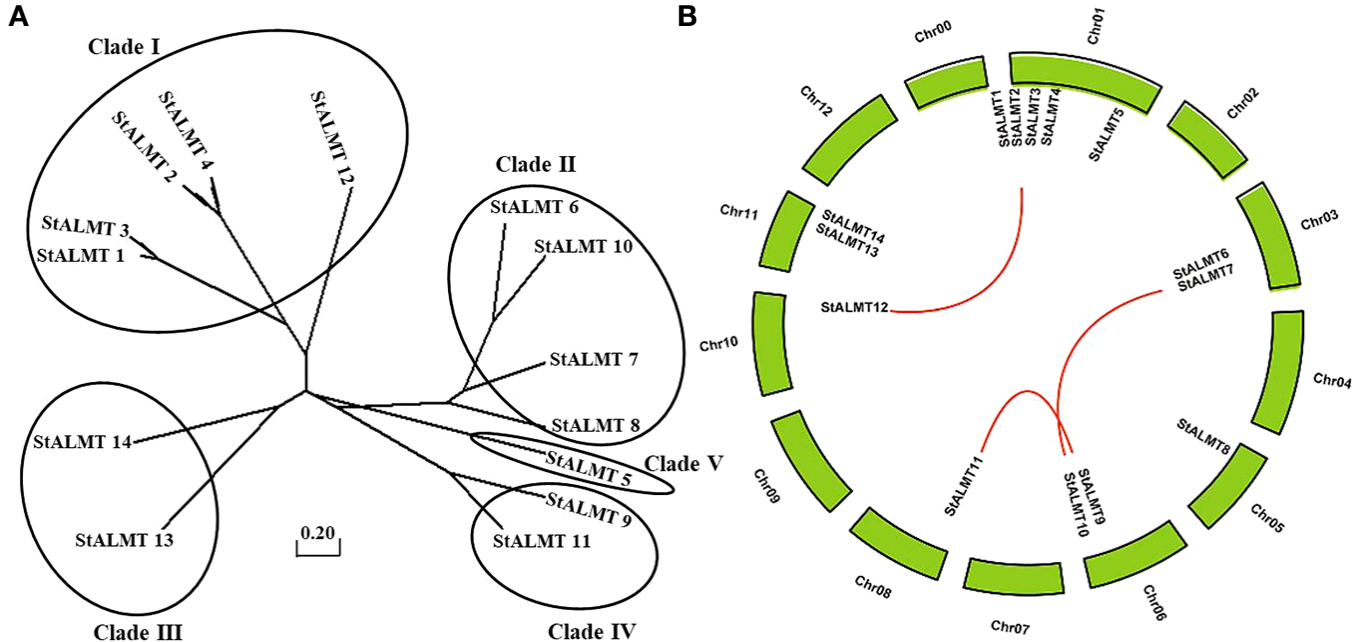
Phylogenetic and syntenic analysis of the potato StALMT genes. **(A)** The phylogenetic tree of the ALMT family of proteins in the potato (*Solanum tuberosum* L.) was obtained from the *Solanum tuberosum* genome v4.03. Amino acid sequence was aligned using the ClustalW program of the MEGA software. **(B)** Syntenic relationships of the *StALMT* genes. Potato chromosomes are displayed as green lines. The putative orthologous *StALMT* genes of the potato are symbolized by red lines.

In the tomato, 16 SlALMT proteins can be classified into four clades (Sasaki et al., 2016). To elucidate the functional and evolutionary relationship of the StALMT family, we obtained a phylogenetic tree of ALMT family proteins of the potato (**Supplementary Table S3**), and 14 members of the StALMT family can be classified into five clades (**Figure 4A**). The results showed that the potato genome contains 14 *ALMT* genes with seven members in clade II, including *StALMT6*, *StALMT7*, *StALMT8*, and *StALMT10*. Interestingly enough, *StALMT6* (PGSC0003DMG400000543) and *StALMT10* (PGSC0003DMG400027029) are highly homologous in clade II and should belong to the same group (**Figure 4A**). Collinearity analysis in the genome revealed three collinear gene pairs, namely, *StALMT1/StALMT12*, *StALMT6/StALMT10*, and *StALMT9/StALMT11* (**Figure 4B**).

### 3.4 Collinearity and phylogenetic analysis of ALMT genes in the potato

**Figure 5:**
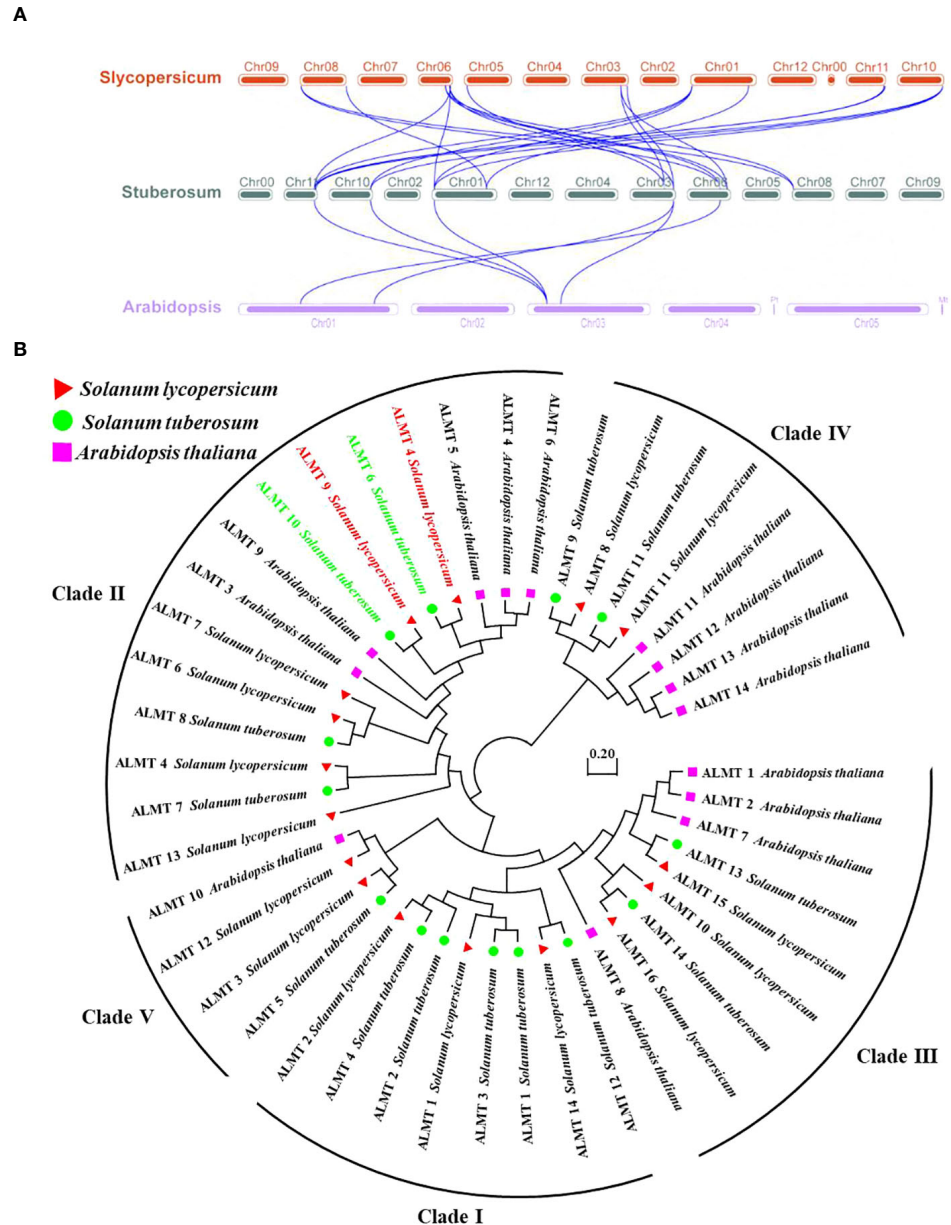
Collinearity and phylogenetic analysis of aluminum-activated malate transporter (ALMT) in the tomato, the potato, and Arabidopsis. **(A)** Collinearity analysis of *ALMT* genes in the tomato, the potato, and Arabidopsis. Chromosomes of the tomato, the potato, and Arabidopsis are represented as red, dark green, and purple, respectively. The blue curve indicates *ALMT* genes with collinearity. **(B)** Phylogenetic analysis of the ALMT proteins from the tomato, the potato, and Arabidopsis using the complete protein sequences. The neighbor-joining (NJ) tree was constructed using the MEGA software with the pairwise deletion option, and 1,000 bootstrap replicates were used to assess tree reliability. The ALMT proteins of the tomato, the potato, and Arabidopsis are represented as red triangles, green circles, and purple squares, respectively.

To further study the syntenic relationship between the potato *StALMT* gene and other plant genomes, two comparative syntenic maps were constructed using the TBtools software. The results showed that potatoes had 6 and 22 orthologous gene pairs with Arabidopsis and tomatoes, respectively, and more than one syntenic gene pair was identified for some of the *StALMT* family members. For instance, *StALMT7* and Arabidopsis AT3G18440.1 and AT1G68600.1 were associated, while *StALMT13* was orthologous with tomato Solyc01g007080, Solyc06g074100, Solyc10g081890, and Solyc11g068970. This means that the evolutionary relationship between the potato and tomato genomes is closer and has a wider range of collinearity compared to Arabidopsis. We further examined the evolutionary history of the *StALMT* family and its subfamily classification; we acquired a total of 30 ALMT protein sequences from Arabidopsis and tomato for alignment (**Figure 5A**).

In the tomato, SlALMT4 and SlALMT5 genes are expressed mainly in the vascular bundles of the fruit during development, and both proteins possess malate efflux functions (Sasaki et al., 2016). Furthermore, researchers performed a genome-wide association study (GWAS) with 272 tomato accessions, revealing that tonoplast-localized SlALMT9 was involved in determining the malic acid content of the fruit (Ye et al., 2017). To investigate the evolutionary relationships of ALMT in different plants, the amino acid sequences from the tomato (16 members of the ALMT protein family), the potato (14 members of the ALMT protein family), and Arabidopsis (14 members of the ALMT protein family) were used to generate the phylogenetic tree. The analysis showed that the 44 ALMT proteins can be classified into five clades, with potato StALMT6 and StALMT10 proteins being highly homologous to tomato SlALMT4 (Solyc03g096820) and SlALMT9 (Solyc06g072920) proteins, respectively (**Figure 5B**, **Supplementary Figure S1**).

### 3.5 Phylogenetic, conserved protein motif, cis-regulatory element, and gene structure analysis of the StALMT family

**Figure 6:**
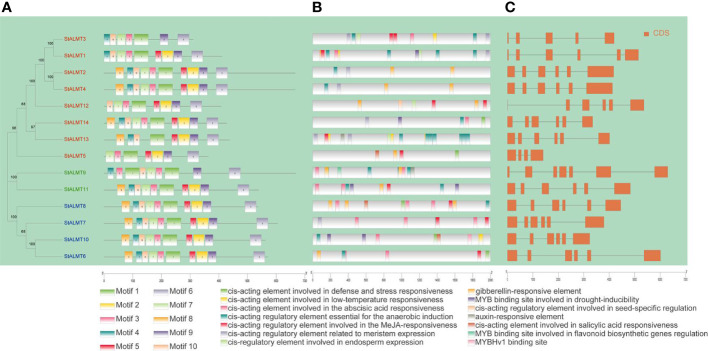
Analysis of the cis-regulatory element distribution in the ALMT promoters. **(A)** Protein sequence motifs in ALMT marked by different colors. **(B)** The number of cis-elements in different categories. **(C)** Structural analysis showing the different StALMT genes containing 4–6 CDS regions.

The phylogenetic tree constructed using the protein sequences of 14 *StALMT* genes showed that these genes can be divided into two categories (**Figure 6A**). Conservative motif analysis showed that the *StALMT* family contained 10 different types of motifs, among which motif 6 and motif 9 were identified in all family members. Unlike eight members in class I, the distance between motif 6 and motif 9 of six members in class II is larger. Cis-acting elements can interact with transcription factors to regulate the growth and development of plants and play an important role in responding to various biotic or abiotic stresses. Therefore, we extracted sequences 2 kb upstream (5′) of the 14 *StALMT* genes to identify cis-acting elements using the PlantCARE web service. The results showed that in addition to a large number of light-responsive core promoter element and common cis-acting elements, many promoter regions of *StALMT* family members contained numerous cis-acting originals of hormone response (**Figure 6B**). At the same time, some genes contain cis-acting originals related to stress responses such as drought. Interestingly, *StALMT10* also contains a cis-acting element, i.e., the MYB-binding site involved in the regulation of flavonoid biosynthetic genes. Abundant regulatory elements indicate the diversity of potential gene functions. Furthermore, structural analysis shows that different *StALMT* genes contain 4–6 CDS regions (**Figure 6C**).

### 3.6 StALMT family expression analysis in different tissues

**Figure 7:**
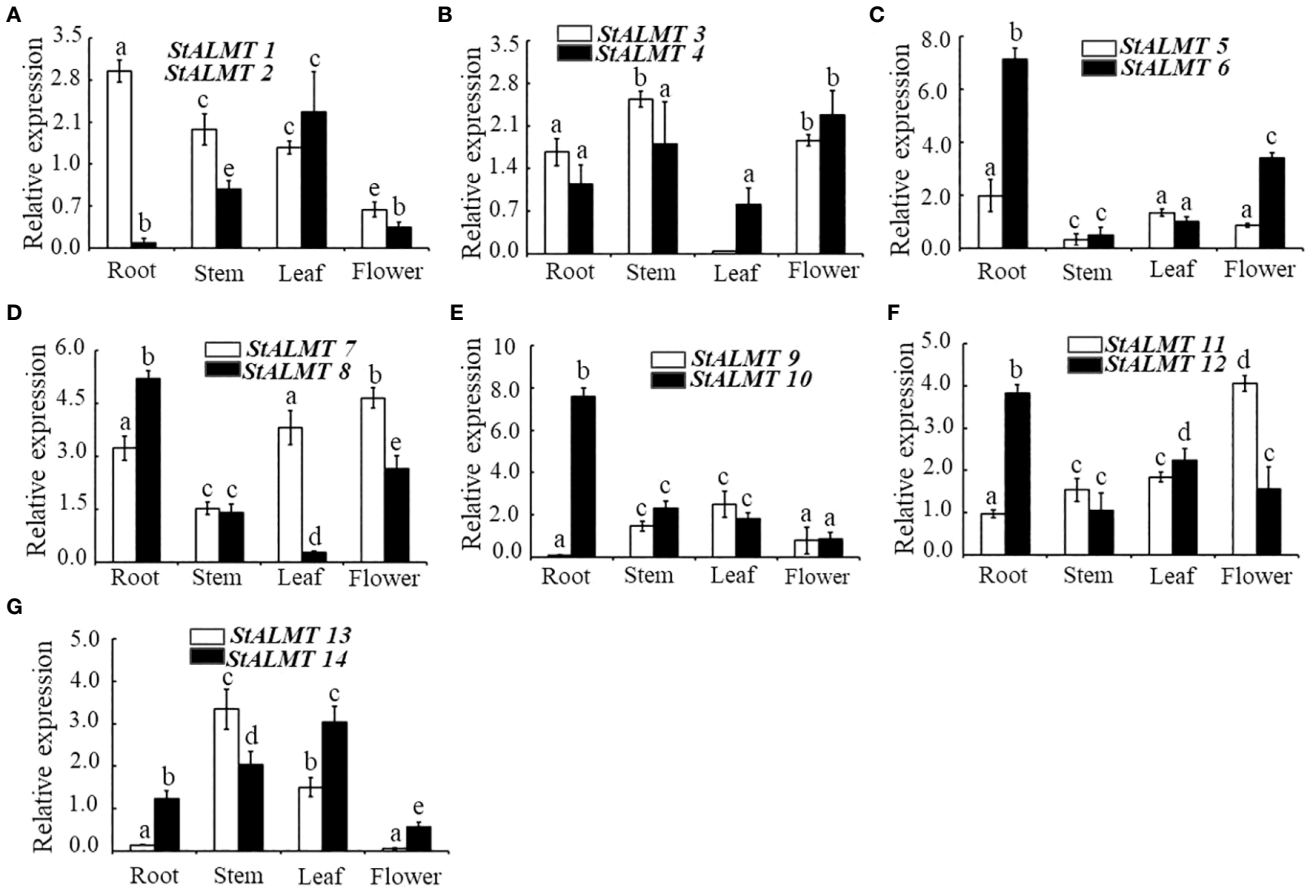
Expression of the *StALMT* family members in different tissues. The expression of **(A)** *StALMT1* and *StALMT2*, **(B)** *StALMT3* and *StALMT4*, **(C)** *StALMT5* and *StALMT6*, **(D)** *StALMT7* and *StALMT8*, **(E)** *StALMT9* and *StALMT10*, **(F)** *StALMT11* and *StALMT12*, and **(G)** *StALMT13* and *StALMT14* in a 7-week-old potato. Actin (PGSC0003DMT400047481) was used as an internal standard. Statistical data are presented as the mean ± standard deviation of three replicate experiments. Different letters indicate significant differences (p < 0.05).

To understand the different tissue expression patterns of the *ALMT* family in the potato, the roots, stems, and leaves of potato cultivar Qingshu No. 9 were analyzed by RT-qPCR. The results showed that *StALMT1*, *StALMT6*, *StALMT8*, *StALMT10*, and *StALMT12* had higher expression in potato roots; *StALMT3*, *StALMT4*, *StALMT13*, and *StALMT14* were highly expressed in stems; *StALMT2*, *StALMT8*, *StALMT10*, *StALMT12*, *StALMT13*, and *StALMT14* had extremely low expression in leaves; and *StALMT3*, *StALMT4*, *StALMT7*, *StALMT8*, and *StALMT11* were highly expressed in flowers. The expression of *StALMT6*, *StALMT8*, *StALMT10*, and *StALMT12* in leaves was lower than that in roots (**Figure 7**). These results suggested that *StALMT* genes have different functions in different tissues.

### 3.7 Expression analysis of StALMTs in the potato in case of Al toxicity

**Figure 8:**
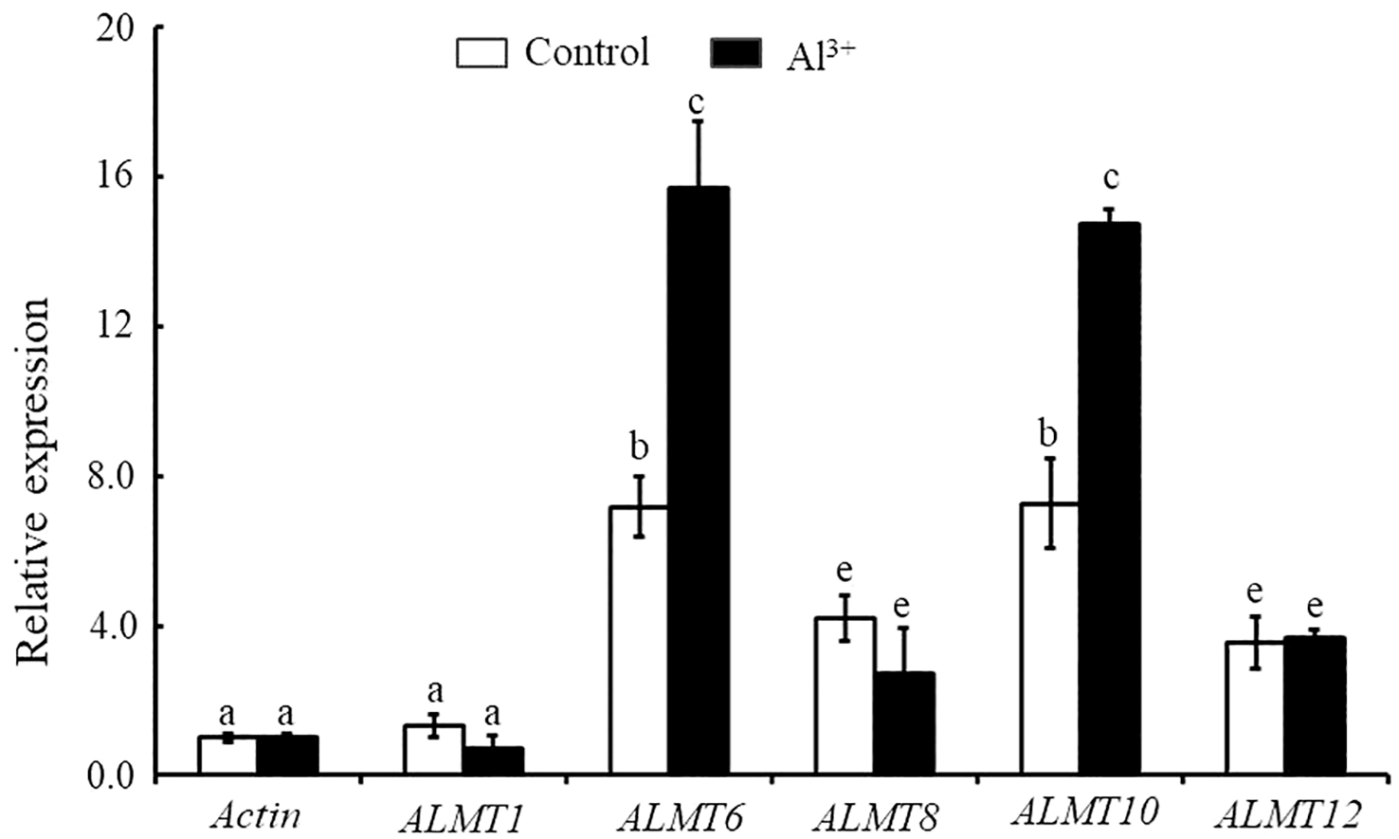
The expression of *StALMT1*, *StALMT6*, *StALMT8*, *StALMT10*, and *StALMT12* genes related to Al^3+^ toxicity in the potato after treatment. Actin (PGSC0003DMT400047481) was used as an internal standard. Statistical data are presented as the mean ± standard deviation of three replicate experiments. Different letters indicate significant differences (p < 0.05).

In the current study, we found that *StALMT1*, *StALMT6*, *StALMT8*, *StALMT10*, and *StALMT12* had higher expression in potato roots (**Figure 7**). In addition, phylogenetic analysis showed that StALMT6 was highly homologous to SlALMT4, and StALMT10 to SlALMT9 (**Figure 5B**, **Supplementary Figure S1**). Therefore, we assessed the expression patterns of *StALMT1*, *StALMT6*, *StALMT8*, *StALMT10*, and *StALMT12* due to Al toxicity. The experimental results showed that under Al ion stress, the expression levels of *StALMT6* and *StALMT10* genes were significantly upregulated, while *StALMT1*, *StALMT8*, and *StALMT12* genes did not show significant changes. Compared with the control group, the relative expression level of *StALMT6* increased 2-fold, and the relative expression level of *StALMT10* increased 1.5-fold. These findings indicate that potatoes respond to Al ion toxicity by upregulating the expression of *StALMT6* and *StALMT10* genes under Al ion stress (**Figure 8**).

### 3.8 The growth of OE-StALMT6 and OE-StALMT10 after Al^3+^ treatment

**Figure 9:**
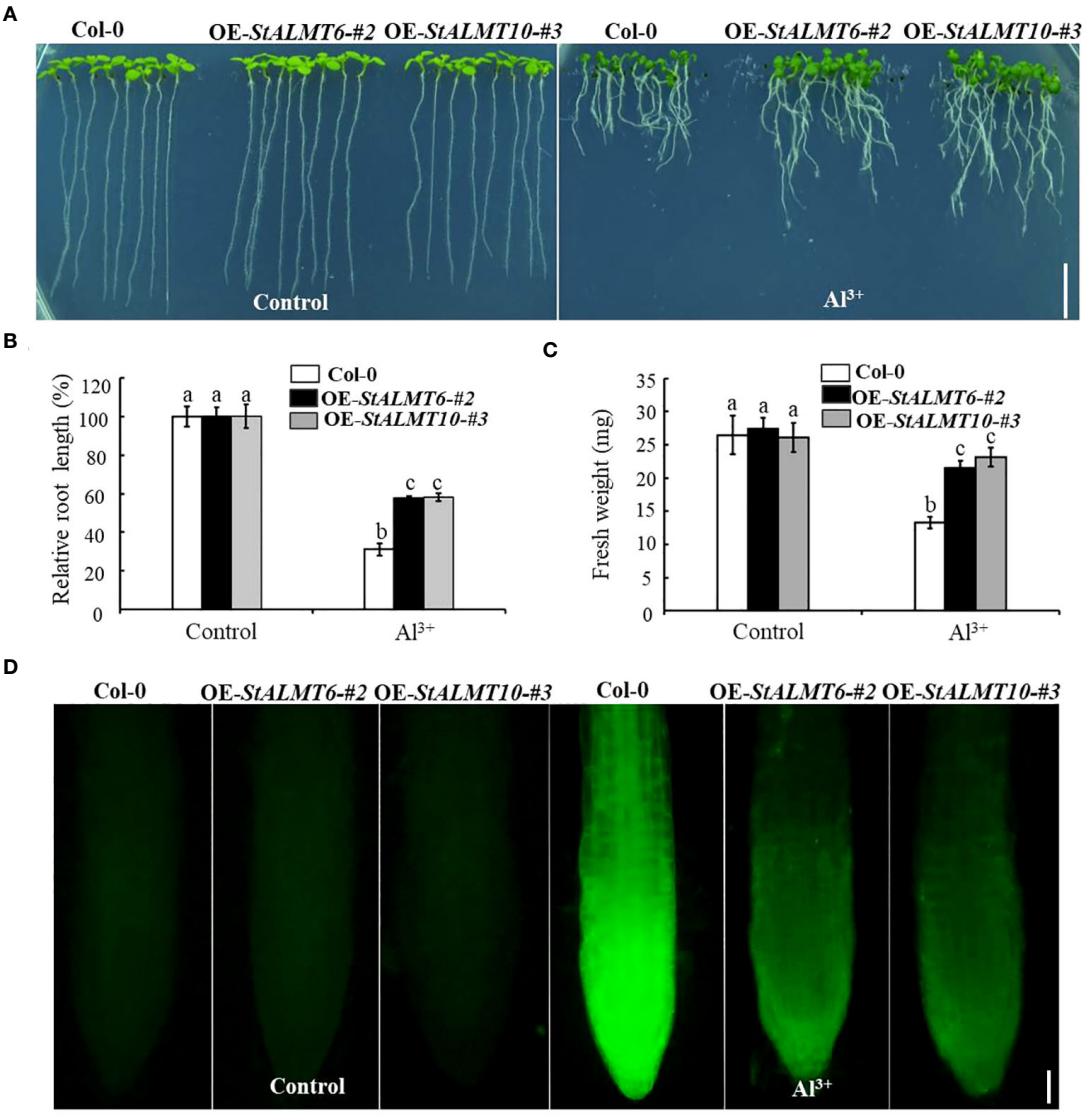
The growth phenotype of Col-0, OE-*StALMT6-#2*, and OE-*StALMT10-#3* after Al^3+^ treatment. **(A)** The growth phenotype of Col-0, OE-*StALMT6-#2*, and OE-*StALMT10-#3* after Al^3+^ treatment (0 and 300 µM), which was sourced from Al_2_(SO_4_)_3_. **(B)** Relative root length statistics. **(C)** Fresh weight statistics. Pictures were taken on the sixth day after cultivation. Scale bar = 1.0 cm. Statistical data are presented as the mean ± standard deviation of three replicate experiments. Different letters indicate significant differences (p < 0.05). **(D)** Morin fluorescence staining of the root tips of Col-0, OE-*StALMT6-#2*, and OE-*StALMT10-#3* after 0 and 300 µM Al^3+^ treatment for 6 **(h)** Scale bar = 100 μm.

To explore whether *StALMT6* and *StALMT10* respond to Al toxicity, we overexpressed the *StALMT6* and *StALMT10* genes of the potato in Arabidopsis (Col-0) by gene transformation and subsequently screened the progeny for overexpression lines of these genes (**Supplementary Figure S3**). The Col-0, OE-*StALMT6-#2*, and OE-*StALMT10-#3* plants grew comparably on the normal medium. Under higher Al^3+^ conditions (300 µM), *StALMT6* and *StALMT10* overexpressors displayed longer primary roots than Col-0 did, indicating that *StALMT6* and *StALMT10* are involved in Al resistance in the potato (**Figure 9A**). The fresh weight measurements show and are consistent with the degree of difference in root length probably because the main toxic site of Al toxicity is the root, so the difference between the two seedlings is not obvious, and the shoots of seedlings account for a greater proportion of the overall fresh weight (**Figures 9B, C**).

The fluorescent reagent morin can emit green fluorescence after binding to small amounts of Al, which can be observed under a fluorescence microscope; morin specifically binds Al in the plant cytoplasm (Eggert, 1970; Oh et al., 2014). To further clarify the role of *StALMT6 a*nd *StALMT10* in Al resistance in the potato, we examined Al accumulation in the roots of Col-0, OE-*StALMT6-#2*, and OE-*StALMT10-#3* using morin fluorescence analysis. Without Al^3+^ treatment, the Col-0, OE-*StALMT6-#2*, and OE-*StALMT10-#3* roots have the same bright fluorescence intensity. However, after being exposed to 300 µM Al^3+^, Col-0 has more fluorescence intensity than OE-*StALMT6-#2* and OE-*StALMT10-#3* (**Figure 9D**). These results suggest that *StALMT6-#2* and *StALMT10-#3* may play a role in Al resistance in the potato.

### 3.9 Relationship between malate secretion and Al toxicity

**Figure 10:**
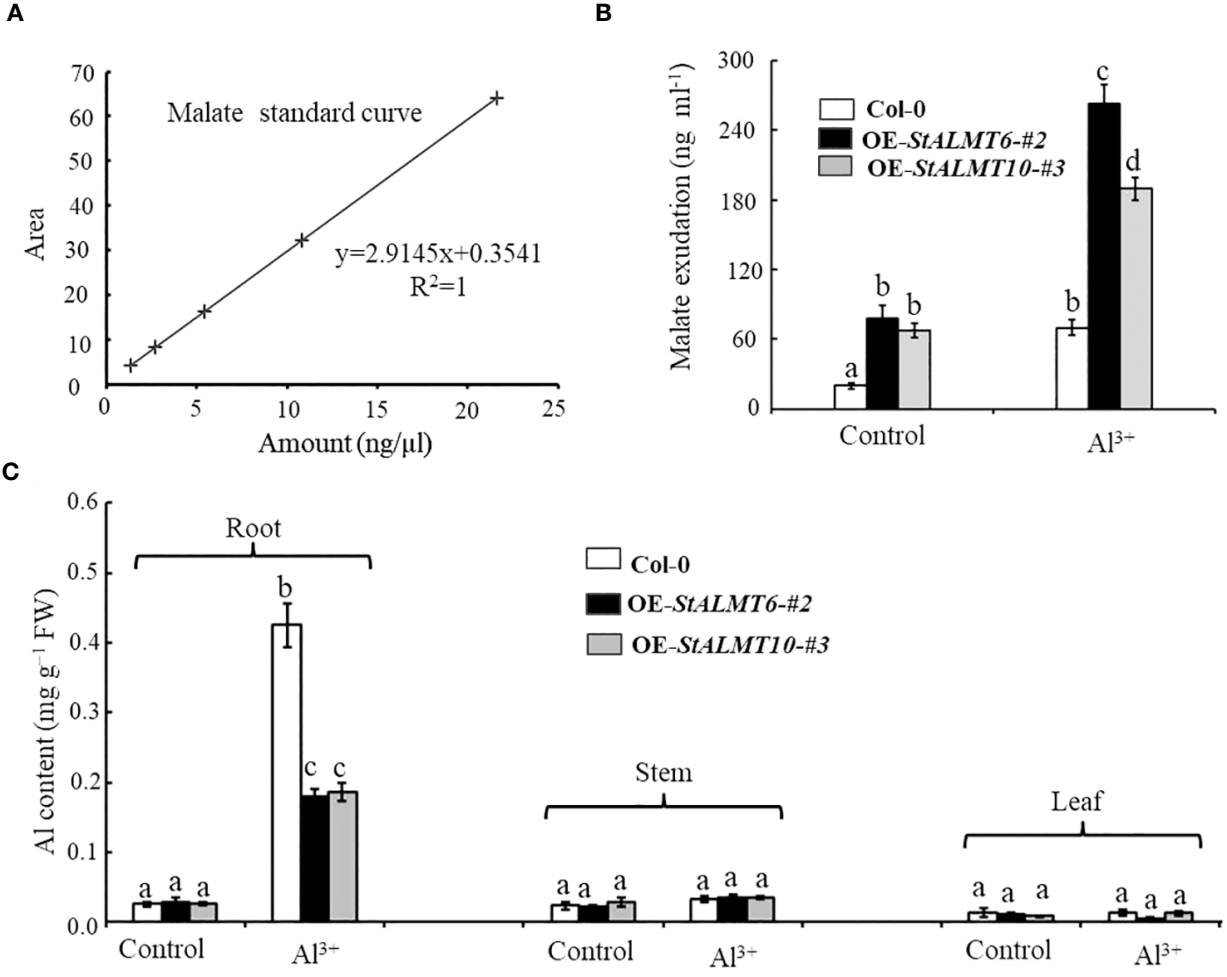
The overexpression plants excrete malate in response to Al toxicity. **(A)** Standard curves of malate detected by HPLC. **(B)** Malate exudate from the 3-week-old Col-0, OE-*StALMT6-#2*, and OE-*StALMT10-#3* incubated in the normal solutions (Control) and solution containing 300 μM Al^3+^ for 72 (h) **(C)** Al contents in the root and shoot of Col-0, OE-*StALMT6-#2*, and OE-*StALMT10-#3* on day 3 after transferring to the solutions without or with 300 μM Al^3+^. Data are means ± SD of three independent plant samples in three replicates. Different letters indicate significant differences (p < 0.05).

In the study of external rejection mechanism of plant resistance to Al toxicity, the secretion of malate efflux from plant roots has been considered to be one of the important mechanisms of resistance to Al toxicity (Deng et al., 2022; Wang et al., 2023). In order to evaluate whether there was a correlation between malate efflux and its Al toxicity, we examined the malate content in Col-0, OE-*StALMT6-#2*, and OE-*StALMT10-#3* root exudates using HPLC. First, we obtained standard curves of malate (**Figure 10A**). In the absence of Al treatment, Col-0, OE-*StALMT6-#2*, and OE-*StALMT10-#3* roots released the same amount of malate. However, after being exposed to 300 µM Al^3+^, OE-*StALMT6-#2* and OE-*StALMT10-#3* roots secreted more malate than Col-0 roots did (**Figure 10B**). These results indicate that the Al^3+^ toxicity growth phenotype OE-*StALMT6-#2* and OE-*StALMT10-#3* may be related to enough malate secretion.

In order to investigate the mechanism of Al resistance in plants overexpressing StALMT6 and StALMT10, the Al content in the different plant lines was measured using ICP-MS in this study. After treatment with 300 µM Al^3+^ for 3 days, the Al content in the roots of both Col-0 and overexpression plants showed a significant increase. However, the Al accumulation in the Col-0 plants was much higher compared to the overexpression plants (**Figure 10C**). In contrast, there was no significant change in the Al content in the stems and leaves of both Col-0 and overexpression plants, and the Al contents were similar after treatment with the same concentration of Al^3+^ (**Figure 10C**). These results suggest that the Al-resistant growth phenotype observed in the OE-*StALMT6-#2* and OE-*StALMT10-#3* plants may be attributed to reduced Al^3+^ accumulation.

## 4 Discussion

A total of 14 potato genome *StALMT* family members are consistent with corresponding members in the publicly reported Arabidopsis (Kovermann et al., 2007). More than 12 *ALMT* family members in peach and Chinese plum (Lin et al., 2018), 16 *ALMT* family members in strawberry (Lin et al., 2018) and tomato (Sasaki et al., 2016), 21 family members in apple (Lin et al., 2018), 25 family members in European pear (Lin et al., 2018), 27 family members in Chinese white pear (Lin et al., 2018), 30 family members in common tobacco (Zhang et al., 2020), and 34 family members in soybean have been identified (Peng et al., 2018). In our study, we found 14 *ALMT* family members in the potato that are distributed on seven different chromosomes (**Figure 1**); this is similar to the findings reported on the chromosomal distribution of ALMT family members in other species (Ma et al., 2018). The N-terminus of potato ALMT family members contains 3–7 transmembrane domains (**Supplementary Table S1**), which is consistent with previous reports on apple (6–7 transmembrane domains) (Ma et al., 2018) and tobacco (4–6 transmembrane domains) (Zhang et al., 2020).

The structure of proteins plays a crucial role in their function and activity. The cryo-EM structure of AtALMT1 bound to malate under neutral or acidic pH conditions has been resolved in Arabidopsis (Wang et al., 2022). By using bioinformatics online tools to analyze and predict the amino acid sequences of StALMT6 and StALMT10, this study successfully obtained their tertiary structure models (**Figure 3**). These predictive results provide important clues for further understanding the functions and regulatory mechanisms of these two genes. In addition, this study also used a protein structure prediction software to predict the secondary structure of the amino acid sequences of StALMT6 and StALMT10. The results showed that the secondary structure of these two genes mainly consists of α-helices and β-sheets (**Figure 2**). This is consistent with the secondary structure features of many studied transport proteins, indicating that StALMT6 and StALMT10 may have similar structures and functions. These predictive results provide valuable information for further studying the functions and regulatory mechanisms of these two genes. Future research can validate these predictive results through experiments and further explore the relationship between the structure and function of StALMT6 and StALMT10.

The tomato and potato belong to the *Solanaceae* family; a total of 16 members of the *SlALMT* family have been identified, and two genes (*SlALMT4* and *SlALMT9*) function to generate the malate efflux (Sasaki et al., 2016; Ye et al., 2017). We used bioinformatics to perform collinearity and phylogenetic analysis on the *ALMT* genes in the potato, the tomato, and Arabidopsis (**Figure 2**). The phylogenetic evolutionary analysis of StALMT6 and StALMT10 proteins with the tomato ALMT family proteins revealed 98% homology between StALMT6 and SlALMT4 (Sasaki et al., 2016), and StALMT10 and SlALMT9 (Ye et al., 2017) (**Supplementary Figure S1**). Furthermore, qRT-PCR showed that *StALMT1*, *StALMT6*, *StALMT8*, *StALMT10*, and *StALMT12* had higher expression in potato roots (**Figure 7**). These results suggest that *StALMT6* and *StALMT10* may play important roles in Al resistance and malate secretion in the potato.

Upregulation of the *AtALMT1* gene promotes the efflux of malate in Arabidopsis cells, thereby alleviating the toxic effects of Al ions (Ding et al., 2013). In this study, we analyzed the gene expression of potatoes under Al ion stress, with a particular focus on the expression of the *StALMT6* and *StALMT10* genes. The experimental results showed that the expression levels of the *StALMT6* and *StALMT10* genes were significantly upregulated under Al ion stress, while the *StALMT1*, *StALMT8*, and *StALMT12* genes did not show significant changes (**Figure 8**). Compared to the control group, the relative expression levels of the *StALMT6* and *StALMT10* genes increased more than 2-fold. These findings indicate that potatoes respond to Al ion toxicity by upregulating the expression of the *StALMT6* and *StALMT10* genes.

This regulatory mechanism can minimize the damage of Al ions to the plant root system, thus enhancing the plant’s resistance to Al toxicity. Morin fluorescence happens through the reaction of the dye with Al, and this method is usually used to evaluate whether the plants are resistant to Al toxicity (Rincón and Gonzales, 1992; Eticha et al., 2005). In order to study the Al toxicity function of the *StALMT6* and *StALMT10* genes, this study successfully transferred the *StALMT6* and *StALMT10* genes into *A. thaliana* plants, obtaining overexpression lines. The presence of the *StALMT6* and *StALMT10* genes in the transgenic plants was confirmed by RT-PCR technique, laying the foundation for further research on the function of these two genes under Al toxicity conditions (**Supplementary Figure S3**). Furthermore, this study compared the phenotypes of wild-type plants with transgenic lines that overexpressed the *StALMT6* and *StALMT10* genes under Al toxicity conditions. The experimental results clearly demonstrated that, under identical Al toxicity conditions, the transgenic lines exhibited significantly reduced root inhibition compared to the wild-type plants (**Figure 9A**). However, the statistical analysis of root length and weight aligned with the growth phenotype (**Figures 9B, C**), suggesting that overexpression of the *StALMT6* and *StALMT10* genes can effectively alleviate the inhibitory effects of Al toxicity on the roots. In addition, by measuring the fluorescence intensity of Al ions, it was found that the accumulation of Al ions in the roots of the *StALMT6* and *StALMT10* gene overexpression lines under Al toxicity conditions was significantly lower than that in wild-type plants (**Figures 9D**, **10C**). This suggests that the overexpression of the *StALMT6* and *StALMT10* genes can inhibit the accumulation of Al ions in plants, thereby reducing the damage caused by Al toxicity.

The secretion of organic acids (OAs), such as citrate, malate, and oxalate, in response to Al toxicity is a well-documented mechanism that helps plants resist Al toxicity (Yang et al., 2019; Ofoe et al., 2023) We analyzed the malate content in root exudates of Col-0, OE-*StALMT6-#2*, and OE*-StALMT10-#3* using HPLC (**Figures 10A, B**). In the absence of Al treatment, all three lines released similar amounts of malate (**Figure 10B**). However, after exposure to 300 µM Al^3+^, OE-*StALMT6-#2* and OE-*StALMT10-#3* secreted more malate than Col-0 did (**Figure 10B**). In addition, we conducted ICP-MS analysis of Col-0, OE-*StALMT6-#2*, and OE-*StALMT10-#3* to quantify the Al content in various tissues under Al-treated and nontreated conditions. It found that the roots of OE-*StALMT6-#2* and OE-*StALMT10-#3* contained lower levels of Al compared to those of Col-0 after Al treatment, while no significant differences were observed in the stems and leaves (**Figure 10C**). In conclusion, through the study of heterologous overexpression lines of the *StALMT6* and *StALMT10* genes, it was found that the overexpression of these two genes can significantly alleviate the inhibition of Al toxicity on plant roots and reduce the accumulation of Al ions. The findings of the study suggest that StALMT6 and StALMT10 do not function in aluminum ion transport but instead regulate aluminum ion uptake by promoting the excretion of organic acids. These results provide an important theoretical basis for further research on the molecular mechanisms of Al toxicity and breeding improvement.

In summary, we have performed a systematic analysis and functional validation of the potato *ALMT* family. A total of 14 *StALMT* genes were identified, which were unevenly distributed on seven chromosomes. Phylogenetic analysis showed that the *StALMT* family can be divided into two classes and five subfamilies, among which *StALMT6* and *StALMT10* are highly homologous genetically to the tomato for evolutionary purposes. Expression analysis showed that most *StALMTs* were expressed mainly in the roots, where the expression changes of *StALMT6* and *StALMT10* were most pronounced under Al toxicity conditions. Arabidopsis overexpression plants transgenic for *StALMT6* and *StALMT10* genes were found to display a growth phenotype resistant to Al toxicity. These results not only provide useful information for illuminating the functions of the *ALMT* gene family in potato but also establish a basis for understanding the molecular mechanisms involved in the response of some of their members to abiotic stresses, especially Al toxicity.

## Data availability statement

The original contributions presented in the study are included in the article/**Supplementary Material**, further inquiries can be directed to the corresponding author/s.

## Author contributions

YA: Writing – review & editing, Writing – original draft. FZ: Writing – original draft, Conceptualization, Formal Analysis, Methodology, Resources, Software, Writing – review & editing. SJ: Formal Analysis, Writing – original draft, Methodology, Writing – review & editing. QL: Formal Analysis, Writing – original draft. ZS: Data curation, Writing – review & editing. YY: Data curation, Formal Analysis, Methodology, Writing – original draft. SY: Funding acquisition, Visualization, Writing – review & editing. ZN: Resources, Writing – review & editing. MC: Writing – original draft, Investigation.

## References

[B1] An Y. Xia X. Jing T. Zhang F. (2022). Identification of gene family members and a key structural variation reveal important roles of OVATE genes in regulating tea (Camellia sinensis) leaf development. Front. Plant Sci. 13. doi: 10.3389/fpls.2022.1008408 PMC9539550 36212328

[B2] Chen W. Tang L. Wang J. Zhu H. Jin J. Yang J.. (2022). Research advances in the mutual mechanisms regulating response of plant roots to phosphate deficiency and Al toxicity. Int. J. Mol. Sci. 23 (3), 1137. doi: 10.3390/ijms23031137 35163057 PMC8835462

[B3] Chen Z. C. Yokosho K. Kashino M. Zhao F. J. Yamaji N. Ma J. F. (2013). Adaptation to acidic soil is achieved by increased numbers of cis-acting elements regulating ALMT1 expression in Holcus lanatus. Plant J. 76 (1), 10–23. doi: 10.1111/tpj.12266 23773148

[B4] Clough S. J. Bent A. F. (1998). Floral dip: a simplified method for Agrobacterium-mediated transformation of Arabidopsis thaliana. Plant J. 16 (6), 735–743. doi: 10.1046/j.1365-313x.1998.00343.x 10069079

[B5] Collins N. C. Shirley N. J. Saeed M. Pallotta M. Gustafson J. P. (2008). An ALMT1 gene cluster controlling aluminum tolerance at the Alt4 locus of rye (Secale cereale L). Genetics 179 (1), 669–682. doi: 10.1534/genetics.107.083451 18493079 PMC2390642

[B6] Deng X. Li Y. Yao K. Qiao J. Wang J. Lin J. (2022). Advances in the mechanism of plant adaptation to acid aluminum stress. Sheng Wu Gong Cheng Xue Bao 38 (8), 2754–2766. doi: 10.13345/j 36002408

[B7] Ding Z. J. Yan J. Y. Xu X. Y. Li G. X. Zheng S. J. (2013). WRKY46 functions as a transcriptional repressor of ALMT1, regulating aluminum-induced malate secretion in Arabidopsis. Plant J. 76 (5), 825–835. doi: 10.1111/tpj.12337 24118304

[B8] Eggert D. A. (1970). The use of morin for fluorescent localization of aluminum in plantissues. Stain Technol. 45 (6), 301–303. doi: 10.3109/10520297009067806 5490089

[B9] Eticha D. Stass A. Horst W. J. (2005). Localization of aluminium in the maize root apex: can morin detect cell wall-bound aluminium? J. Exp. Bot. 56 (415), 1351–1357. doi: 10.1093/jxb/eri136 15797941

[B10] Gasteiger E. Hoogland C. Gattiker A. Duvaud S. E. Wilkins M. R. Appel R. D.. (2005). “Protein identification and analysis tools on the ExPASy server,” in The Proteomics Protocols Handbook. Ed. Walker J. M. (Totowa, NJ: Humana Press), 571–607.

[B11] Gruber B. D. Ryan P. R. Richardson A. E. Tyerman S. D. Ramesh S. Hebb D. M.. (2010). HvALMT1 from barley is involved in the transport of organic anions. J. Exp. Bot. 61 (5), 1455–1467. doi: 10.1093/jxb/erq023 20176888 PMC2837267

[B12] Hoekenga O. A. Maron L. G. Piñeros M. A. Cançado G. M. A. Shaff J. Kobayashi Y.. (2006). AtALMT1, which encodes a malate transporter, is identified as one of several genes critical for aluminum tolerance in Arabidopsis. Proc. Natl. Acad. Sci. U.S.A 103 (25), 9738–9743. doi: 10.1073/pnas.0602868103 16740662 PMC1480476

[B13] Horton P. Park K. J. Obayashi T. Fujita N. Harada H. Adams-Collier C. J. (2007). Nakai K. WoLF PSORT: protein localization predictor. Nucleic Acids Res. 35 (Web Server issue), W585–W587. doi: 10.1093/nar/gkm259 17517783 PMC1933216

[B14] Kelley L. A. Mezulis S. Yates C. M. Wass M. N. Sternberg M. J. (2015). The Phyre2 web portal for protein modeling, prediction and analysis. Nat. Protoc. 10 (6), 845–858. doi: 10.1038/nprot.2015.053 25950237 PMC5298202

[B15] Kobayashi Y. Sugimoto M. Lakshmanan V. Iuchi S. Kobayashi M. Bais H. P.. (2013). Characterization of the complex regulation of AtALMT1 expression in response to phytohormones and other inducers. Plant Physiol. 162 (2), 732–740. doi: 10.1104/pp.113.218065 23624855 PMC3668066

[B16] Kochian L. Pifieros M. Liu J. Magalhaes J. (2015). Plant adaptation to acid soils:the molecular basis for crop aluminum resistance. Annu. Rev. Plant Biol. 66, 571–598. doi: 10.1146/annurev-arplant-043014-114822 25621514

[B17] Kovermann P. Meyer S. Hörtensteiner S. Picco C. Scholz-Starke J. Ravera S.. (2007). The Arabidopsis vacuolar malate channel is a member of the ALMT family. Plant J. 52 (6), 1169–1180. doi: 10.1111/j.1365-313X.2007.03367 18005230

[B18] Li N. Wang J. Wang B. Huang S. Hu J. Yang T.. (2021). Identification of the carbohydrate and organic acid metabolism genes responsible for brix in tomato fruit by transcriptome and metabolome analysis. Front. Genet. 12. doi: 10.3389/fgene.2021.714942 PMC8446636 34539743

[B19] Ligaba A. Katsuhara M. Ryan P. R. Shibasaka M. Matsumoto H. (2006). The BnALMT1 and BnALMT2 genes from rape encode aluminum-activated malate transporters that enhance the aluminum resistance of plant cells. Plant Physiol. 142 (3), 1294–1303. doi: 10.1104/pp.106.085233 17028155 PMC1630743

[B20] Ligaba A. Maron L. Shaff J. Kochian L. Piñeros M. (2012). Maize ZmALMT2 is a root anion transporter that mediates constitutive root malate efflux. Plant Cell Environ. 35 (7), 1185–1200. doi: 10.1111/j.1365-3040.2011.02479 22211473

[B21] Lin X. Xin Q. Ming Y. Z. Shao Z. (2018). Genome-Wide analysis of aluminum-activated malate transporter family genes in six rosaceae species, and expression analysis and functional characterization on malate accumulation in Chinese white pear. Plant Sci. 274, 451–465. doi: 10.1016/j.plantsci.2018.06.022 30080635

[B22] Livak K. J. Schmittgen T. D. (2001). Analysis of relative gene expression data using real-time quantitative PCR and the 2(-Delta Delta C(T)) Method. Methods 25 (4), 402–408. doi: 10.1006/meth.2001.1262 11846609

[B23] Lu J. Du J. Tian L. Li M. Zhang X. Zhang S.. (2021). Divergent response strategies of CsABF facing abiotic stress in tea plant: perspectives from drought-tolerance studies. Front. Plant Sci. 12. doi: 10.3389/fpls.2021.763843 PMC8635920 34868162

[B24] Ma B. Yuan Y. Gao M. Qi T. Li M. Ma F. (2018). Genome-wide identification, molecular evolution, and expression divergence of aluminum-activated malate transporters in apples. Int. J. Mol. Sci. 19 (9), 2807. doi: 10.3390/ijms19092807 30231490 PMC6163302

[B25] Magalhaes J. V. Piñeros M. A. Maciel L. S. Kochian L. V. (2018). Emerging pleiotropic mechanisms underlying aluminum resistance and phosphorus acquisition on acidic soils. Front. Plant Sci. 9. doi: 10.3389/fpls.2018.01420 PMC6168647 30319678

[B26] Ofoe R. Thomas R. H. Asiedu S. K. Wang-Pruski G. Fofana B. Abbey L. (2023). Aluminum in plant: Benefits, toxicity and tolerance mechanisms. Front. Plant Sci. 13. doi: 10.3389/fpls.2022.1085998 PMC9880555 36714730

[B27] Oh M. W. Roy S. K. Kamal A. H. Cho K. Cho S. W. Park C. S.. (2014). Proteome analysis of roots of wheat seedlings under aluminum stress. Mol. Biol. Rep. 41 (2), 671–681. doi: 10.1007/s11033-013-2905-8 24357239

[B28] Peng W. Wu W. Peng J. Li J. Lin Y. Wang Y.. (2018). Characterization of the soybean GmALMT family genes and the function of GmALMT5 in response to phosphate starvation. J. Integr. Plant Biol. 60 (3), 216–231. doi: 10.1111/jipb.12604 29045000

[B29] Piñeros M. A. Cançado G. M. Maron L. G. Lyi S. M. Menossi M. Kochian L. V. (2008). Not all ALMT1-type transporters mediate aluminum-activated organic acid responses: the case of ZmALMT1 - an anion-selective transporter. Plant J. 53 (2), 352–367. doi: 10.1111/j.1365-313X.2007.03344 18069943

[B30] Qin L. Tang L. H. Xu J. S. Zhang X. H. Zhu Y. Zhang C. R.. (2022). Cryo-EM structure and electrophysiological characterization of ALMT from Glycine max reveal a previously uncharacterized class of anion channels. Sci. Adv. 8 (9), eabm3238. doi: 10.1126/sciadv.abm3238 35235352 PMC8890709

[B31] Qin Z. Chen S. Feng J. Chen H. Qi X. Wang H.. (2022). Identification of aluminum-activated malate transporters (ALMT) family genes in hydrangea and functional characterization of HmALMT5/9/11 under aluminum stress. PeerJ 10, e13620. doi: 10.7717/peerj.13620 35769137 PMC9235816

[B32] Ribeiro A. P. Vinecky F. Duarte K. E. Santiago T. R. das Chagas Noqueli Casari R. ,. A. Hell A. F.. (2021). Enhanced aluminum tolerance in sugarcane: evaluation of SbMATE over-expression and genome-wide identification of ALMTs in Saccharum spp. BMC Plant Biol. 21 (1), 300. doi: 10.1186/s12870-021-02975-x 34187360 PMC8240408

[B33] Rincón M. Gonzales R. A. (1992). Aluminum partitioning in intact roots of aluminum-tolerant and aluminum-sensitive wheat (Triticum aestivum L.) cultivars. Plant Physiol. 99 (3), 1021–1028. doi: 10.1104/pp.99.3.1021 16668966 PMC1080579

[B34] Sasaki T. Ariyoshi M. Yamamoto Y. Mori I. C. (2022). Functional roles of ALMT-type anion channels in malate-induced stomatal closure in tomato and Arabidopsis. Plant Cell Environ. 45 (8), 2337–2350. doi: 10.1111/pce.14373 35672880

[B35] Sasaki T. Tsuchiya Y. Ariyoshi M. Nakano R. Ushijima K. Kubo Y.. (2016). Two Members of the Aluminum-Activated Malate Transporter Family, SlALMT4 and SlALMT5, are Expressed during Fruit Development, and the Over-expression of SlALMT5 Alters Organic Acid Contents in Seeds in Tomato (Solanum lycopersicum). Plant Cell Physiol. 57 (11), 2367–2379. doi: 10.1093/pcp/pcw157 27615796

[B36] Sasaki T. Yamamoto Y. Ezaki B. Katsuhara M. Ahn S. J. Ryan P. R.. (2004). A wheat gene encoding an aluminum-activated malate transporter. Plant J. 37 (5), 645–653. doi: 10.1111/j.1365-313x.2003.01991.x 14871306

[B37] Sharma T. Dreyer I. Kochian L. Piñeros M. A. (2016). The ALMT family of organic acid transporters in plants and their involvement in detoxification and nutrient security. Front. Plant Sci. 7. doi: 10.3389/fpls.2016.01488 PMC5047901 27757118

[B38] Wang Z. Liu L. Su H. Guo L. Zhang J. Li Y.. (2020). Jasmonate and aluminum crosstalk in tomato: Identification and expression analysis of WRKYs and ALMTs during JA/Al-regulated root growth. Plant Physiol. Biochem. 154, 409–418. doi: 10.1016/j.plaphy.2020.06.026 32650255

[B39] Wang P. Wan N. Horst W. J. Yang Z. B. (2023). From stress to responses: Aluminium-induced signalling in the root apex. J. Exp. Bot. 74 (5), erac516. doi: 10.1093/jxb/erac516 36609593

[B40] Wang J. Yu X. Ding Z. J. Zhang X. Luo Y. Xu X.. (2022). Structural basis of ALMT1-mediated aluminum resistance in Arabidopsis. Cell Res. 32 (1), 89–98. doi: 10.1038/s41422-021-00587-6 34799726 PMC8724285

[B41] Xu X. Pan S. Zhang S. Mu B. Ni D. Zhang P.. (2011). Genomesequence and analysis of the tuber crop potato. Nature 475 (7355), 189–195. doi: 10.1038/nature10158 21743474

[B42] Xu M. Gruber B. D. Delhaize E. White R. G. James R. A. You J.. (2015). The barley anion channel, HvALMT1, has multiple roles in guard cell physiology and grain metabolism. Physiol. Plant 153 (1), 183–193. doi: 10.1111/ppl.12234 24853664

[B43] Yang J. L. Fan W. Zheng S. J. (2019). Mechanisms and regulation of aluminum-induced secretion of organic acid anions from plant roots. J. Zhejiang Univ Sci. B. 20 (6), 513–527. doi: 10.1631/jzus.B1900188 31090277 PMC6568218

[B44] Ye J. Wang X. Hu T. Zhang F. Wang B. Li C.. (2017). An InDel in the promoter of Al-ACTIVATED MALATE TRANSPORTER9 selected during tomato domestication determines fruit malate contents and aluminum tolerance. Plant Cell. 29 (9), 2249–2268. doi: 10.1105/tpc.17.00211 28814642 PMC5635988

[B45] Ye J. Wang X. Wang W. Yu H. Ai G. Li C.. (2021). Genome-wide association study reveals the genetic architecture of 27 agronomic traits in tomato. Plant Physiol. 186 (4), 2078–2092. doi: 10.1093/plphys/kiab230 34618111 PMC8331143

[B46] Zhang H. Li Z. F. Xu G. Y. (2020). Identification and expression analysis of ALMT gene family in Nicotiana tabacum. Tobacco Sci. Technol. 53 (5), 1–9. doi: 10.16135/j.issn1002-0861.2019.0079

[B47] Zhang L. Wu X. X. Wang J. F. Qi C. D. Wang X. Y. Wang G.. (2018). BoALMT1, an Al-induced malate transporter in cabbage, enhances aluminum tolerance in Arabidopsis thaliana. Front. Plant Sci. 8. doi: 10.3389/fpls.2017.02156 PMC5787101 29410672

[B48] Zhang F. Yan X. Y. Han X. B. Tang R. J. Chu M. L. Yang Y.. (2019). A defective vacuolar proton pump enhances aluminum tolerance by reducing vacuole sequestration of organic acids. Plant Physiol. 181 (2), 743–761. doi: 10.1104/pp.19.00626 31350362 PMC6776860

[B49] Zheng S. J. (2010). Crop production on acidic soils: overcoming aluminium toxicity and phosphorus deficiency. Ann. Bot. 106 (1), 183–184. doi: 10.1093/aob/mcq134 20570831 PMC2889811

