# Supplementary figures and images for: Identification of the ALMT gene family in the potato (Solanum tuberosum L.) and analysis of the function of StALMT6/10 in response to aluminum toxicity
Source: Front Plant Sci. 2023 Nov 20;14:1274260. doi: 10.3389/fpls.2023.1274260 (PMC10694233; doi:10.3389/fpls.2023.1274260)

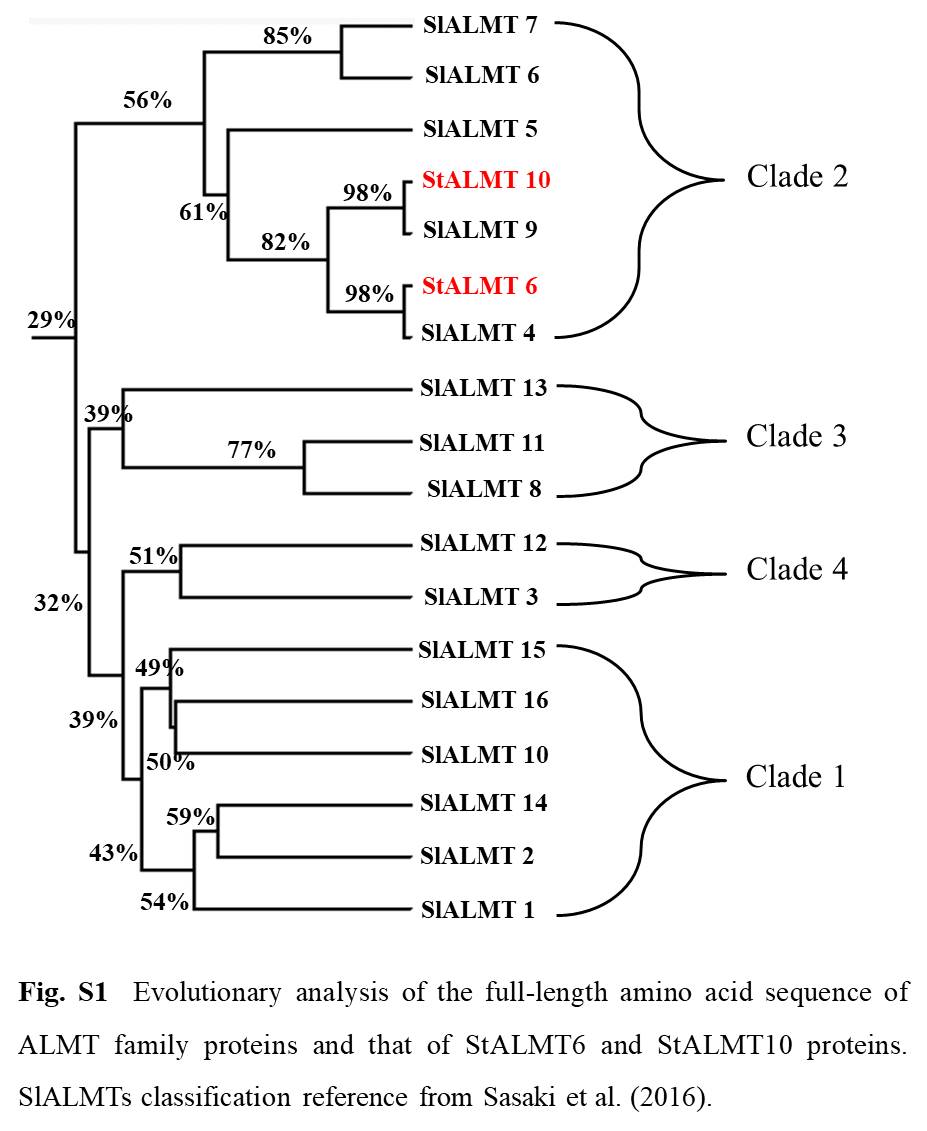

Supplement: Supplementary file 1 [file Image_1.jpeg]

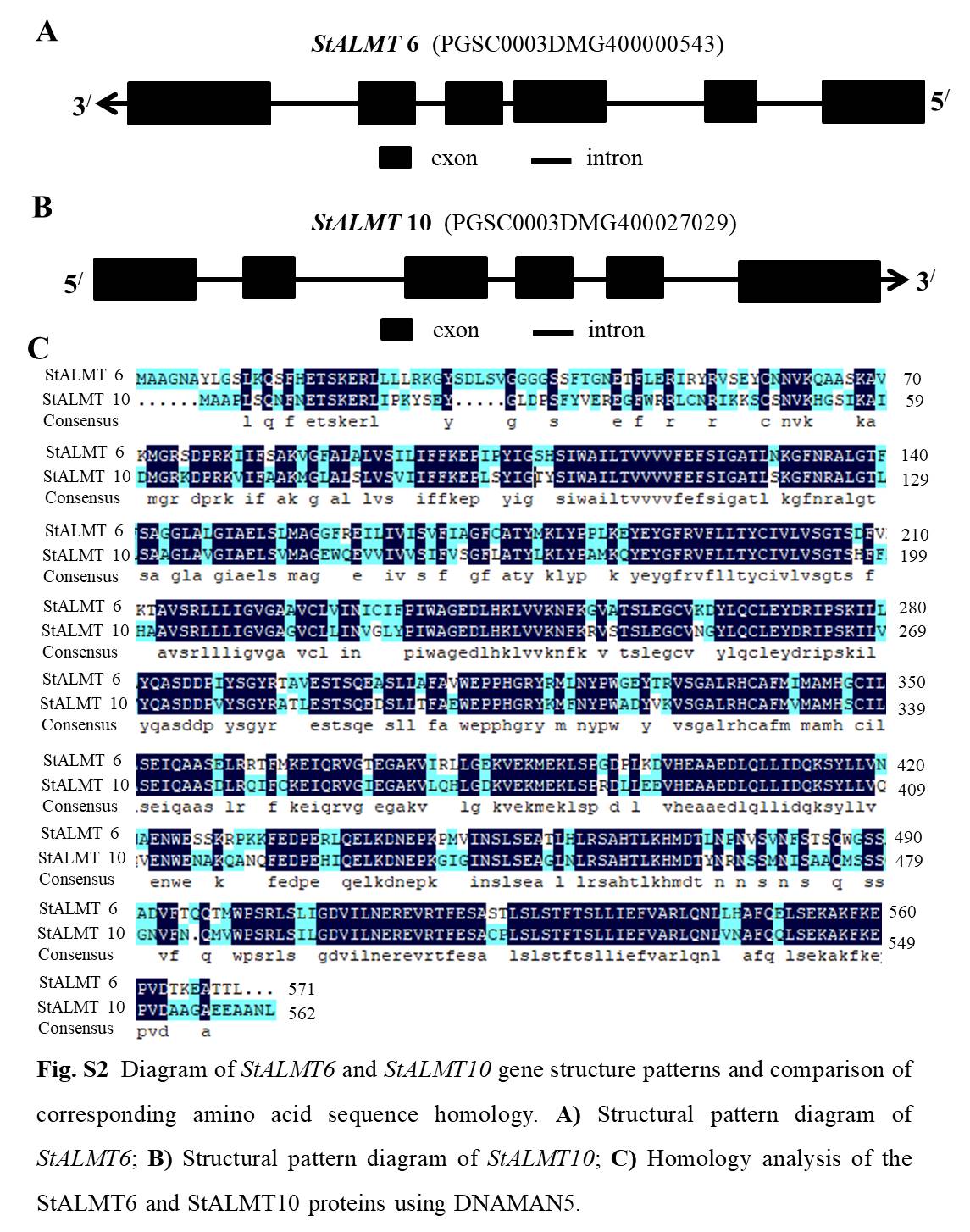

Supplement: Supplementary file 2 [file Image_2.jpeg]

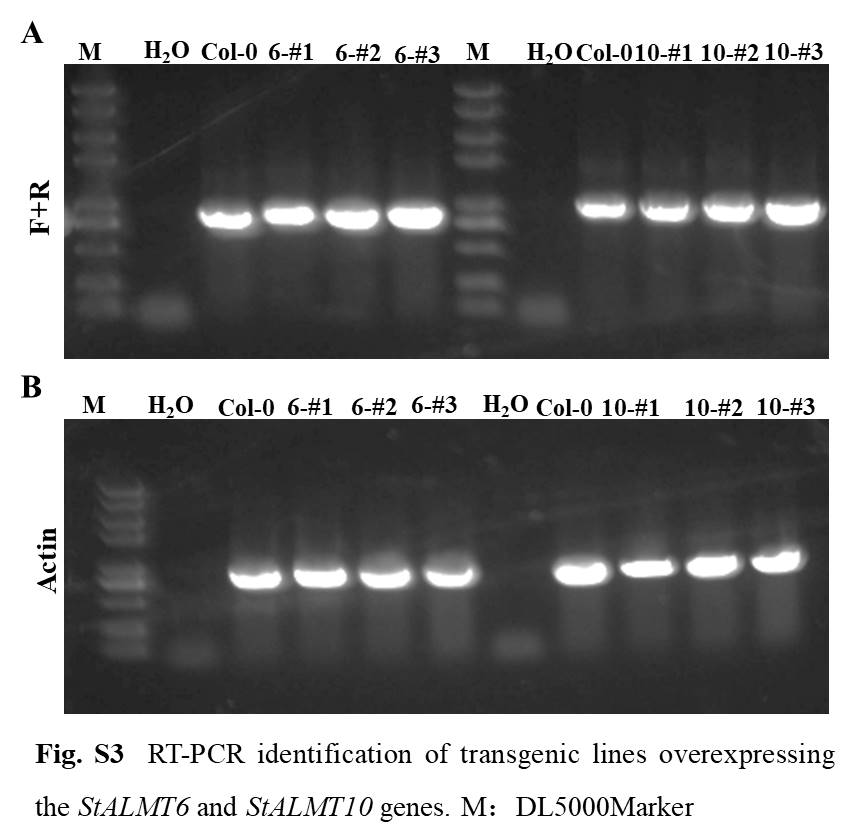

Supplement: Supplementary file 3 [file Image_3.jpeg]
