# Supplementary material for: Identification of the ALMT gene family in the potato (Solanum tuberosum L.) and analysis of the function of StALMT6/10 in response to aluminum toxicity
Source: Front Plant Sci. 2023 Nov 20;14:1274260. doi: 10.3389/fpls.2023.1274260 (PMC10694233; doi:10.3389/fpls.2023.1274260)
Supplement: Supplementary file 4 [file Table_1.docx]

**Table S1** Molecular characteristics of StALMT genes in potato.

| Gene ID | Gene name | | N-amino acids | | Molecular weight | Theoretical pI | Transmembrane domain | Subcellular localization |
| --- | --- | --- | --- | --- | --- | --- | --- | --- |
| PGSC0003DMG400039398 | | *StALMT*1 | | 410 | 45126.37 | 7.56 | 3 | Cyto |
| PGSC0003DMG400036570 | | *StALMT*2 | | 665 | 74500.05 | 5.98 | 5 | Plas |
| PGSC0003DMG400035191 | | *StALMT*3 | | 310 | 34173.96 | 8.78 | 3 | Vacu |
| PGSC0003DMG400037538 | | *StALMT*4 | | 665 | 74640.05 | 6.20 | 5 | Plas |
| PGSC0003DMG400000121 | | *StALMT*5 | | 362 | 40304.34 | 5.97 | 3 | Cyto/E.R./Mito |
| PGSC0003DMG400000543 | | *StALMT*6 | | 571 | 63611.68 | 8.21 | 5 | Plas |
| PGSC0003DMG400005713 | | *StALMT*7 | | 604 | 68383.67 | 6.24 | 5 | Plas/ E.R. |
| PGSC0003DMG400012973 | | *StALMT*8 | | 537 | 60694.20 | 7.91 | 6 | Plas/ E.R |
| PGSC0003DMG400002104 | | *StALMT*9 | | 667 | 74278.56 | 5.97 | 7 | Plas |
| PGSC0003DMG400027029 | | *StALMT*10 | | 562 | 62849.50 | 6.23 | 5 | Plas/ Vacu |
| PGSC0003DMG400020518 | | *StALMT*11 | | 538 | 60401.87 | 8.73 | 5 | Plas/ E.R. |
| PGSC0003DMG400028146 | | *StALMT*12 | | 406 | 44608.71 | 7.17 | 4 | Chlo |
| PGSC0003DMG400019625 | | *StALMT*13 | | 437 | 48636.96 | 8.96 | 5 | Plas/Golg/Vacu |
| PGSC0003DMG400018568 | | *StALMT*14 | | 427 | 47335.41 | 8.98 | 5 | Plas/ Vacu |
