# Supplementary material for: Identification of the ALMT gene family in the potato (Solanum tuberosum L.) and analysis of the function of StALMT6/10 in response to aluminum toxicity
Source: Front Plant Sci. 2023 Nov 20;14:1274260. doi: 10.3389/fpls.2023.1274260 (PMC10694233; doi:10.3389/fpls.2023.1274260)
Supplement: Supplementary file 5 [file Table_2.docx]

**Table S2.** Primers used in this study

| **Primers** | **Sequence 5′-3′** | **Purpose** |
| --- | --- | --- |
| St -Actin-F | GGGATGGAGAAGTTTGGTGGTGG | RT-qPCR |
| St-Actin-R | CTTCGACCAAGGGATGGTGTAGC |  |
| St- ALMT1-F | TATGTCGGTGGCGTCGATAC |  |
| St- ALMT1-R | CCGCGGCATTCACAATTTCA |  |
| St- ALMT2-F | ATCGCGTGTCATGGATTGTG |  |
| St- ALMT2-R | TGCCGGCAACAGACTCTTCT |  |
| St- ALMT3-F | TGGTCATTCGACCAGTGTGG |  |
| St- ALMT3-R | ACTTAGTTGGTGCCAAAGATTCC |  |
| St- ALMT4-F | TCCTGGACTTTGTTCCCACC |  |
| St- ALMT4-R | AGGCGGCGACATACTCTTCT |  |
| St- ALMT5-F | TGCTGTTTGATAAATGACTGGATGG |  |
| St- ALMT5-R | TCAACGCGATAGCCTGACAC |  |
| St- ALMT6-F | TCAGTGGTGCACTACGACAC |  |
| St- ALMT6-R | AGGATCTCCGGGGCTTAGTT |  |
| St- ALMT7-F | GCACCTCCCGAAAGAAGACA |  |
| St- ALMT7-R | CCACTGACCCCAACTTCTCC |  |
| St- ALMT8-F | GGCTTCTCATCAAGATCAACACA |  |
| St- ALMT8-R | TGGAGTGAAACAACAACATCCA |  |
| St- ALMT9-F | AGGCTCTACCATTTGCAGCC |  |
| St- ALMT9-R | AGCAGCATTTCAGTTCCCTTC |  |
| St- ALMT10-F | ACACGGTTCGATCAAGGCAA |  |
| St- ALMT10-R | GTGAGGATGGCCCAAATGGA |  |
| St- ALMT11-F | TTGGGCTGTCATGACTGTGG |  |
| St- ALMT11-R | ATGTACGTCGCTGTAGCACC |  |
| St- ALMT12-F | ATGACACAACCGTCGTCCTC |  |
| St- ALMT12-R | TCAATCAGTACGGACGCCAC |  |
| St- ALMT13-F | CTGTGTGGGCTGGTGAAGAT |  |
| St- ALMT13-R | AAATTGTCCATGCCCTGGCT |  |
| St- ALMT14-F | AGGTGTTGCAGCTCATAGGC |  |
| St- ALMT14-R | TTATCGAGCACCACAGGGTC |  |
| St-ALMT6-F1 | ATGCATGGATGGCAGCAGGCAATGCATATCT | Vector construction |
| St-ALMT6-R1 | CGGACTAGTTAAAGAGGCAACTACTCTTTGAAC |  |
| St-ALMT10-F1 | ACCATGGATGGCGGCGCCATTGAGCCAAAA |  |
| St-ALMT10-R1 | GACTAGTCTACAAGTTAGCTGCCTCCTCTGC |  |
| St-ALMT6-F2 | TCAGCATAGGTGCAACGCTT | Identification of haploid |
| St-ALMT6-R2 | AGTTGCGCAAAATCCTGCAA |  |
| St-ALMT10-F2 | AGGGCCACTTTGGAGTCAAC |  |
| St-ALMT10-R2 | CCATGACCATGAAAGCGCAA |  |
