# Supplementary material for: Identification of the ALMT gene family in the potato (Solanum tuberosum L.) and analysis of the function of StALMT6/10 in response to aluminum toxicity
Source: Front Plant Sci. 2023 Nov 20;14:1274260. doi: 10.3389/fpls.2023.1274260 (PMC10694233; doi:10.3389/fpls.2023.1274260)
Supplement: Supplementary file 6 [file Table_3.docx]

**Table S3.** Amino acid sequences of ALMTs in potato

| **Gene ID and Name** | **Amino acid sequences** |
| --- | --- |
| PGSC0003DMG400039398（StALMT1） | MAVLTVMVAFEYTAGATISKCLNIAFATALGVSLGIGAKYLAKFCGKEGEPIILGCLVFILGALGTFTRFYPHMQRRYDYGCMFSVATFSLVTVSGDRYLELVKQRISTIMVSVATVMAISLVIRPVWAGKDLHNLIIANLEKLASFLDGFESEYFQAIGEGSKDKEKGFLEALKNVLGSKATEESLANFAWWEPANGPFRFNNPGKEYLKIGNLGRDCACHLHALSGHLKSKSKAPTEFHKRTEEAYKRIITKSSNALKCLALSIKTATQPPSSTGEPDPYNAKFAIDELRAALLITTRTISEEDTIDVIMAMSVASILIDVTRCVDEISKAVGELSIKTRFQKEDKKKDNSTSIKVLEKLPAPRLQIIHREIVNAAVKEEENPIDAIKGEHVVCEIHVIEEEIKAEGK |
| PGSC0003DMG400036570 (StALMT2) | MEIDSTNQENLSVFTKWWNKLKDFPRKFQIKIGKIAKNTKQIGKDDPRKIWHAFKVGLALTLVSLFYYTEPLFHNFDEPVMWAVLTVVVTFEFTAGATISKSINRGIGTALAGAFGLGAKYLAELIGREGPNPIVLGVLVFIVGALGTFTRFYPHIKRRYDYGTMIFVLTFCLVAVSGYRSENIFQLAHRRISTILVGVFTVMIISMIIRPVWAGEDLHKLASTNLEKLATYLEGFGSEYFHMSEIKSVEGNNNNEKGFHEAFLSILGSKAIEESLANLALWEPPHGAFKFNHPWKQYLKIGNLVRKCACHLLALSSHIYSKSQAPNEFERRTEEACKKMIMESKKALKELALSIKTMAQPISSIRNTKNVIDDLKLTLGISKTFFRYDESRVMDCVPTASIMSLLISVTKCVDEISEAIEELSSKARFEKKSLLPATTSRSHRSQILHRGTVNPIVEDEVDGGDFVKIEIGDNVEPKGKVVVEEVNPIVEDDVDGGDFVTIEIGDNVESKGKVVVEEVNQMSNQISAKKEESFVIWIHGSTAMAIVEEMKTPKKIRDFMDLVDIKLYNSTQFFESTEGVLTSLEGVNQVSNPLAVTEGEPVVIGICGNTTTTKVEEMEMSEKKVELTYLANIKFDNSIQFLESILMTVTTTVEEMKTAEKKETL |
| PGSC0003DMG400035191 (StALMT3) | MAVLTVMVAFEYTAGATISKCLNIAFATALGVSLGIGAKYLAEFCGKVGEPIILGFLVFILGALGTFTRFYPHMQRRYDYGCMFSVATFSLVTVSGDRYLELVKHRISTIMVSVATVMVISLVIRPVWAGKDLHNLIIANLEKLASFLDGFESEYFQAIGEDSKDKEKGFLEAFKSVLGSKATEESLAPTKFHKRIEEACKGIITKSSNALKCLTLSIKTRTQPPSSTGEPDPYNAKFAIDELRAVLLITTKTISEEDTIDVITAMSVASILIDVTRCVDEISKAVGELSIKARFQKEEKRRILLRQSRR |
| PGSC0003DMG400037538 (StALMT4) | MEIDSTNQENLSIFTKWWNKLKDFPRKFQKKIEKIAKNTKQIGKDDPRKIWHAFKVGLALTLVSLFYYTEPLFHNFDQPVMWAVLTVVVTFEFTAGATISKSINRGIGTALAGAFGLGAKYLAELIGREGPNPIILGVLVFIVGALGTFTRFYPHIKRRYDYGTMIFVLTFSLVAVSGYRSENIFQLAHRRISTILIGVFTVMIISMIIRPVWAGEDLHKLASTNLEKLASYLEGFGSEYFHISENKSVEGNNNNEKGFHEVFLSILGSKATEESLANLAWWEPPHGGFKFNHPWKQYLKIGSLVRKCACHLLALSNHINSKSQAPNEFERRTEEACKKMIMESKKALKELALSIKTMAQPISSIRNTKNVIDDLKLTLGTSKTLFQYDESRVLDFVPTASVVSLLISVTKCVDEISEAIKELSSKARFEKKSMSPPPPSRSHWPQILHRGTVNPIVEDEVDGGDLVTIEIGDNVESKRKVVVEEVNPIVEDDVDGGDFVTIEIGDNVESKGKVVVEEVNRVNNQINATKEESFVIWIRGSTAMAIVEEMKTPKKMGDFMDLVDIKLYNSTQFLESSEGVLTVLEGANQVNNPLTVTEGESVVIGIRGNTTTTKVEEMEMTKKKVELTDLTDIKFNNSIQFLESILMTVTTVVEEMKMTEKKETL |
| PGSC0003DMG400000121 (StALMT5) | MDCCLINDWMDVNMFIHVFLAAAAATFSRFIPTIKARFDYGAMIFILTFSLVSVSGYRVDKLVELAHERVSTIAIGASICIFITMILCPVWAGTELHHLISNNLEKLADSLEGYGAENFSVDGSKNVHEKDSSKRLQGYKCVLNSKAAEESMANFARWEPAHGKFNFRHPWKQYLKIGASMRSCAYCIETLHGGINSNTETPEFLKKPLNDVCMRLGTSSSKVLKEMSSMIKTMKKSTKLDILVDEMNSSVEGLQNALKIFPSYQHIPTPDPATEEAPNCTEETSLKPTALSLMEIVQMATLTSLLVEIASRIEGIVEEVKELATQAEFRDESSKKSKQTQTKINGNDGNEHEVTVKTLQQV |
| PGSC0003DMG400000543 (StALMT6) | MAAGNAYLGSLKQSFHETSKERLLLLRKGYSDLSVGGGGSSFTGNETFLERIRYRVSEYCNNVKQAASKAVKMGRSDPRKIIFSAKVGFALALVSILIFFKEPIPYIGSHSIWAILTVVVVFEFSIGATLNKGFNRALGTFSAGGLALGIAELSLMAGGFREILIVISVFIAGFCATYMKLYPPLKEYEYGFRVFLLTYCIVLVSGTSDFVKTAVSRLLLIGVGAAVCLVINICIFPIWAGEDLHKLVVKNFKGVATSLEGCVKDYLQCLEYDRIPSKILLYQASDDPIYSGYRTAVESTSQEASLLAFAVWEPPHGRYRMLNYPWGEYTRVSGALRHCAFMIMAMHGCILSEIQAASELRRTFMKEIQRVGTEGAKVIRLLGEKVEKMEKLSPGDPLKDVHEAAEDLQLLIDQKSYLLVNAENWESSKRPKKFEDPERLQELKDNEPKPMVINSLSEATLHLRSAHTLKHMDTLNPNVSVNFSTSQWGSSADVFTQQTMWPSRLSLIGDVILNEREVRTFESASTLSLSTFTSLLIEFVARLQNLLHAFQELSEKAKFKEPVDTKEATTL |
| PGSC0003DMG400005713 (StALMT7) | MVRKSNSFRKSFEDRRVKERLLSLCNGVDYTELPGFPHSLDYQETSGCCSSFREKFSELWKDWKRVSVKAMEMGRTDPRKIIFSAKMGLALILMSLFIFFKEPAVKELGKYSVWAILTVVVVFEFSIGATLSKGFNRGLGTLSAGGLALAIAELSQLAGDWEEVVIIIGIFMTGFFITYAKQYPAMKPYEYGFRVFLITYCFIMVSGYHTREFIETAVSRFLLIVLGASISLAVNICVYPIWAGEDLHNLVTKNFINIATSLEGCISEYLNCVECKRIPSKILTYQVADDPVYNGYRSAVESISQEEALEAFAVWEPPHGPYKMIKYPWKNYVKVSGALRYCAFMVMALHGCILSEIQAPPERRQVFRNELQRVGTASAKVLRELGEKVKKMEKLGSVDILYEVHEAAEELQKKVDRKSYLFVNAENWEIGTRAMVVDISQELGSLDEDRSLLQHHRSQSETVINIDSILASKSWDNRTCNLASNNNQTTGVTPENTVEKPKFRTTHSLPKDNDALKEVEAGEDEEVESKTYESASALSLATFTSLLIEFVARLQNVVDSFEELSEKAKFKDPMDLSDASQKVGLWSRFRGCIKFWKRESSLPV |
| PGSC0003DMG400012973 (StALMT8) | MVTIKNFFKKASHQDQHNQEKEKLLQHDDDEIKNGCCCFTPLYERFKSFFNNIQDFAKKAIEMGKNDPRKIIFSLKMGFALSFVSLLIFWKKPTDVAQFAIWAILTVLVMFEFTIGATFIKGFNRGLGTFCAGMLAFIFAQLALWAGEREKAVIVVSIFIVAFIGTYLKLYPTMAPYEYGYRVFILTYCILIVAGNRTREYNVAIFTRLALIAVGAGICLMINISVCPIWAGEDLHRLVVKNFMDLATSLEGCINGYLSGVDCDEATNDSDYNGYKSVIESTSREQTLLGFAIWEPPHGRFKMHKNPWRDIVKLSSGLRHCAFMVMALHGCIQSEIQAPPEKRKVFRNELKKVGTNAAKVLRELGTKLEKLEMLNGHENILKEVHETAQNLQKKVDHKSYLLVNSKSWEIGKSNIINNLDDSSSENSSENIPLSSRSLSETAIDIRSLQGNWPQSDQLVAKLTPFKKQNQWPSRLSLVDGEIADTIEMETYLSASALSLATFASLLIEFVARLQNVVDNFEELSQRAEFKESISVKS |
| PGSC0003DMG400002104 (StALMT9) | MEPLFKGIGENAIWAVMTVVVVLEFTTGAMTTYMRFFSHIKKNYDYGVVIFLLTFNLITVSSYRVDSVLKIAHERFYTIAIGCSICLLMSLFIFPIWSGEDLHLSTVAKFDGLAKSIEGATLYKGLNRGLGTLLAGLLAFLIERIANESGHIFHAAFIGTAVFLVGAMTTYMRFFSHIKKNYDYGVVIFLLTFNLITVSSYRVDSVLKIAHERFYTIAIGCSICLLMSLFIFPIWSGEDLHLSTVAKFDGLAKSIEVCINEYFSDGNINQQEEKVKKYSMEMEDPIYKGYKDVLDSKSSDETLTPQSVRVLFKDPCNRLAREVTKTLKELGDSIRNRRKYSPEIPSNHVHEALQDLIDALKSQPRLFLSTNSNTNILLALATLATRQKSGKDFLVSLPSVNNDDPARFGLSHDLMLKEGDKKILRPTLSKNGITSLEFLEALPFAAFASLLVEIVARLDLVIEQVVELGRVAQFKEYSHDDVVINVSCDNNPRVERRELKCCYKASVDGFSATEFHNRSDFKGPCVIIGYTTKAFKFGAFNPEGYRSTDDYYDTFDAFLFYWDEDVEKPIMLPKVGGSGAALFDYARGGPQFGADGLLIGPPLAPVMGGFAGPDTNSGVGDLRQAKSRLGLSYAKRPDGKESLFGDESKAVIDEVLVFCSPQIASLY |
| PGSC0003DMG400027029 (StALMT10) | MAAPLSQNFNETSKERLIPKYSEYGLDPSFYVEREGFWRRLCNRIKKSCSNVKHGSIKAIDMGRKDPRKVIFAAKMGLALSLVSVIIFFKEPLSYIGTYSIWAILTVVVVFEFSIGATLSKGFNRALGTLSAAGLAVGIAELSVMAGEWQEVVIVVSIFVSGFLATYLKLYPAMKQYEYGFRVFLLTYCIVLVSGTSHFFHAAVSRLLLIGVGAGVCLLINVGLYPIWAGEDLHKLVVKNFKRVSTSLEGCVNGYLQCLEYDRIPSKILVYQASDDPVYSGYRATLESTSQEDSLLTFAEWEPPHGRYKMFNYPWADYVKVSGALRHCAFMVMAMHSCILSEIQAASDLRQIFCKEIQRVGIEGAKVLQHLGDKVEKMEKLSPRDLLEEVHEAAEDLQLLIDQKSYLLVQVENWENAKQANQFEDPEHIQELKDNEPKGIGINSLSEAGLNLRSAHTLKHMDTYNRNSSMNISAAQMSSSGNVFNQMVWPSRLSILGDVILNEREVRTFESACPLSLSTFTSLLIEFVARLQNLVNAFQQLSEKAKFKEPVDAAGAEEAANL |
| PGSC0003DMG400020518 (StALMT11) | MVVEMEVNNGENTTNNWVPIMKKLHMNVIGEKMKKIPKLTCRTIWRVGKEDPRRVIHSLKVGISLTLVSLLYLMDPLFKGFGSNAIWAVMTVVVVLEFTAGATLCKGLNRGLGTLLAGSLAFLIEYVATKTGHVFRAIFIGAALFFIGATATYMRFIPYIKKNYDYGVVIFLLTFNLITVSSYRVDNVLKIAHERFYTIAIGCGVCLLMSLLIFPNWSGEDLHNFTAAKFEGLAKSIEACVNEYFSDEEQQKTKENSSDLEDPIYNGYKTVLDSKSFDETLALYASWEPRHSRHCYRFPWQQYVKLGNVLRHFGYTIVALHGCLQTEIQTPRSVRAMFKDPCIRLSGEVAKALKELGDSIRYRRHCSPEILSDHLHEALHDLNKAIKSQPKLFIGSKNNTNKLTLATVVSKGQSGALNSRRVSLSSVKTDTSALLDWKSKLRGGSSTTHENLKLRPTLSKIAITSLEFSEALPFAAFASLLVEIVARLDLVIEEVEELGRIAHFKEYNDDDEHHVVVEIDKKNSKPLPSQLPTQDSGD |
| PGSC0003DMG400028146 (StALMT12) | MNKSATLSKCLNRGFATLTAGALGVGAKYFADLFGKEGEPIVLGILVFTLGALGTFTRFFPHMKRRYDYGILIFVLTFSMVTVSGYRVDKILELAHQRLSTILIGAATCMIVSLIVCPVWAGEDLHKLIYTHLEKLANFLEGFGSEYFIFSENDEIVKASNEGFLGSYKTVLNSKANEEALANFAWWEPGHGSFRLRHPWKQYLKIGVLARECACHLQALSGYFNSKPQVPSEFQKKIEEACTKMCIESSKVLKELAFSIKTMTQPSSSAAEIHLRHSKAAVDDFKSILAATETLLLSNKLDLLEIFPAITVASVLIDVINCIDKISESVEDLSVQAHFKKAKNKEFSSSSPEKPPPQHQLLHRGIVKPVVDVDDVDSGGDFVAIEICGGGAAVAGKAEVNPAVVKK |
| PGSC0003DMG400019625  (StALMT13) | TMEIASMNNDEKPSNFFTKGLLWFKSLHEKIFTKIVGIAKQFKTIAKDDPRRVIHSLKVGLSLTLVSLFYYFQPLYNGFGVSTMWAIMTVVVVFEFSVGATLGKGLNRGMATLIAGALGVGAHYLASATGKVVEPILLGLFVFLQVSISGFRVDEIVDMAHKRISTIFMGASVCVIVSIVVCPVWAGEDLHKLVAQNMEKLGKFLEGFGDEIFKSSEDITESKVTKTSLIEYKSVLNSKNTEETLANFAKWEPGHGQFKYRHPWKQYLKIGGLIRQCACRIDALNAYINSEIKAPEEIREMIKETSMKMSIECGKALKELSKSMRKMNLPISADEHVTNAKNSAKKLNSLLKSRINNWEEINLLQVIPLATIASILTDIVICVEEIGQGVNELASLANFKTTKSSKEIINTNKVMVVEKIELSNNNVVITINEQNMKL |
| PGSC0003DMG400018568 (StALMT14) | MCSFGSNVKKIAKEDPRKLIHSIKVGLAIALVSLFYYFEPLFDYEGFGVSSMWAVLTVVVVFEFSVGATLGKGVNRGLATFLAGSLGVAAHRLASFTGSDKLQPIILGLSVFSVATIATFLRFVPKLKARYDYGILIFILTFSMISVSGYRDPVVLDKAITRVTTILIGGVAAIMFNVVIYPVWAGEDLHNLIATNIEELGISLEGYGTQYFKKIDVKSEEELERISLDEYKCVIYSKASEENLVNFAKWEPHHGKFRYRHPWGQYLKIGDLVRECAIKINALNLDLTSCNMTQEGRKIIQEQCTKMSIECGSALKEIAMSMKTMTLYPTIDSHILKAKASSEKLRSIIRNGSLIEEKELQKLLPSTRIASLMLDIVSNSVEIVDSVNQLATLTKFKILSSKPKRLGSKSRIPSGNIGEAHLVVNVE |
